# Numerical Analysis of BIMs for Stochastic SIR and SIS Models with Variable Contact Diffusion Rates

**DOI:** 10.1007/s00285-026-02392-4

**Published:** 2026-05-14

**Authors:** Henri Schurz, Kursad Tosun

**Affiliations:** 1 Department of Mathematics, Southern Illinois University, 1245 Lincoln Drive, Carbondale, 62901 IL USA; 2 Department of Mathematics, Siena College, 15 Loudon Road, Loudonville, 12211 NY USA

**Keywords:** Stochastic SIR model, Stochastic SIS model, Balanced implicit methods, Dynamic consistency, Stochastic differential equations, Mathematical epidemiology, 92D30, 92D08, 60H10, 60H35, 65C30

## Abstract

Qualitative properties of adequate numerical methods for stochastic differential equations to simulate the stochastic counterparts of some known models with variable contact diffusion rates in mathematical epidemiology are investigated, especially SIS and SIR models with non-constant total population sizes on bounded domains. We show the convergence of numerical approximations based on Balanced Implicit Methods (BIMs) by proving several properties; including positivity, invariance, stability, mean and mean square consistency, local uniform boundedness, mean square Hölder continuity and mean square contractivity. We also present some simulation results for those models with realistic parameters.

## Introduction

Mathematical epidemiology studies the spread of diseases in populations by using tools from mathematics, statistics and computer science. Because of ethical concerns and the nature of diseases, it is difficult to do experiments searching for an effective strategy for the management of diseases. Mathematical models may be preferred. SIR models are the foundation of compartmental epidemiological models. These models divide the population into three classes: *Susceptible* (=S), *Infected* (=I) and *Removed* (=R). If the disease confers no immunity against reinfection then infected individuals return directly to the susceptible class. Such a model is called a SIS model (Brauer and Chávez 2012). In 1927, the simplest SIR model to incorporate the dynamics of the recovered population was introduced by Kermack and McKendrick (1927). Since then, by including more assumptions in the models, their predictive power and applicability improved.

Because infectious diseases are subject to randomness in terms of the nature of transmission and environmental fluctuation, stochastic modeling is appropriate to describe the evolution of an infectious disease, relying on the laws of large numbers. Deterministic models also explore many types of transmission. Frequency-dependent and mass-action dependent transmission are two frequent transmission types used in both deterministic and stochastic models. However, when the population is small or the number of infected individuals is not large, stochasticity can have a major impact (Keeling and Rohani 2008). Other properties that are unique to the stochastic epidemic models include the probability of an outbreak and the eradication of an endemic disease (Allen 2008).

In this paper we present investigations and a justification of numerical methods, which show adequate qualitative behavior for stochastic SIR and SIS models with births and deaths and varying contact and recovery rates. To incorporate stochasticity, noise is introduced directly into related deterministic models (cf. Allen (2010); Gray et al. (2011); Schurz and Tosun (2015); Schurz (2019); Allen (2007); Gard (1988); Chandrasena and Chandrasena (2024)). Our models represent a rather non-parametric approach to the class of stochastic SIR and SIS models with respect to the diffusion terms (i.e., "non-parametric" means that a rather general functional dependence of the diffusion intensities is considered here), in contrast to the standard stochastic models with constant diffusion intensities in the literature (see Beretta et al. (1998); Beretta and Kuang (1998); Carletti (2002); Dalal et al. (2007, yyy); Gray et al. (2011); Jianga et al. (2011); Imhofa and Walcher (2005); Lahrouz et al. (2011a, 2011b); Tornatore et al. (2005)). Most of the previous investigations of those models center around qualitative aspects of associated stochastic dynamic systems. For example, asymptotic stability of exact solutions are studied in Carletti (2002); Chandrasena and Chandrasena (2024); Chandrasena et al. (2025); Lahrouz et al. (2011a, 2011b); Schurz and Tosun (2015); Schurz (2019); Tornatore et al. (2005). Lyapunov functionals to study global properties are constructed and verified in Chandrasena and Chandrasena (2024); Chandrasena et al. (2025); De Leon (2009); Fall et al. (2007); Korobeinikov and Wake (2002); Korobeinikov (2006)). A discrete time SI model with controlled vaccination is found in Chen (2006).

Because exact analytic solutions of stochastic SIS, SIS, SEIV or SEIR models are rarely known one must often rely on numerical methods to derive quantitative results on stochastic SIR and SIS models (and similar models). This paper provides a systematic analysis of appropriate numerical methods built upon balanced implicit methods (BIMs) for those models, revealing many adequate properties of underlying dynamics and their mathematical justification. The crucial problem is to construct convergent numerical methods, which possess the same uniform boundedness properties under quite erratic random noise terms in order to demonstrate some meaningful biologic relevance. In this respect, this paper represents an extension of Schurz (1996, 2007) from bounded domains $\mathbb {D}$ in $\mathbb {R}^1$ to $\mathbb {D}$ in $\mathbb {R}^3$ or $\mathbb {R}^2$. More precisely, we consider the stochastic SIR model introduced in Schurz and Tosun (2015) with disease deaths and variable diffusion rates $F_j$ driven by standard Wiener processes $W_j$

1 $$\begin{aligned} \displaystyle dS= &  \Big (-\beta S I +\mu (K-S)\Big )~dt - S I~ F_1\big (S,I,R\big ) ~dW_1,\nonumber \\ dI= &  \Big (\beta S I-\big (\alpha +\gamma +\mu \big )I\Big )~ dt + S I~ F_1\big (S,I,R\big ) ~dW_1-I~F_2\big (S,I,R\big ) ~dW_2, \nonumber \\ dR= &  \Big (\alpha I - \mu R\Big )~dt+ I~F_2\big (S,I,R\big ) ~dW_2 \end{aligned}$$

where contact rate $\beta$, recovery rate $\alpha$, natural death rate $\mu$ and disease related death rate $\gamma$ are positive. The diffusion rate functions $F_j$’s are locally Lipschitz-continuous on the bounded prism

2 $$\begin{aligned} \mathbb {D}=\Big \{(S,I,R) \in \mathbb {R}^3\!: ~S> 0, ~I \ge 0, ~R > 0, ~S+I+R\le K\Big \} \end{aligned}$$

for all $t \ge 0$. In this model, $\mu K$ represents the number of births (immigrants), where *K* is a carrying capacity and $\beta S I$ represents the number of new infections in unit time. For the total population $N(t)=S(t)+I(t)+R(t)$, we obtain the ordinary differential equation (ODE):

$$\begin{aligned} N'(t)=\mu (K-N(t))-\gamma I(t)~ \end{aligned}$$

by adding the above equations of system (1). Therefore, the population size *N* of SIR model (1) is not constant and may vary in time.

Furthermore, we also take into account a stochastic SIS model with disease deaths and variable diffusion rates, which was suggested and analyzed in Schurz (2019)

3 $$\begin{aligned} \begin{aligned} dS=&\Big (-\beta S I +\mu (K-S)+\alpha I \Big )~dt -S I~F_1(S,I)~dW_1 +I~F_2(S,I)~dW_2, \\ dI=&\Big (\beta S I-(\alpha +\gamma +\mu )I\Big )~ dt + S I ~F_1(S,I)~dW_1 -I~F_2(S,I)~dW_2 \end{aligned} \end{aligned}$$

where the parameters $\alpha$, $\beta$, $\gamma$ and $\mu$ are positive and the diffusion rate functions $F_j$’s are locally Lipschitz-continuous on the bounded triangular domain

4 $$\begin{aligned} \mathbb {D}=\Big \{(S,I) \in \mathbb {R}^2\!: ~S > 0, ~I \ge 0, ~S+I\le K\Big \} \end{aligned}$$

for all $t \ge 0$. Here, similar to the SIR model (1), the population size *N* with $N(t)=S(t)+I(t)$ is not constant for the SIS model (3) and satisfies the ODE $N'(t)=\mu (K-N(t))-\gamma I(t)$ by adding all equations in (3).

The paper is organized by 6 sections. After this introduction, Section 2 presents the main class of balanced implicit methods (BIMs) under investigation, basic concepts of numerical approximations and the relevant $L^2$-convergence notions based on an axiomatic approach (following Schurz (2006)). Section 3 is devoted to the axiomatic numerical analysis of BIMs for the stochastic SIR model (1) with disease deaths and variable diffusion rates. Section 4 is carrying out a similar numerical analysis for the stochastic SIS model (3). Eventually, Sections 5 and 6 report on some numerical experiments and conclusions.

## Balanced Implicit Method and Convergence

The general class of Theta Balanced Implicit Methods (BIMs) (extracted from Schurz (1997)) is defined by

5 $$\begin{aligned} \begin{aligned} X_{n+1}=&\;X_n+ \Big [\Theta _n ~a(X_{n+1},t_{n+1})+ (I_{d\times d}-\Theta _n) ~a(X_{n},t_{n}) \Big ] ~\Delta _n \\&\quad + \sum _{j=1}^m b^j(X_n,t_n) \Delta W_n^j +\sum _{j=0}^m c^j(X_n,t_n)(X_n-X_{n+1})~|\Delta W_n^j| \end{aligned} \end{aligned}$$

with appropriate (bounded) matrices $c^j$ with continuous entries, where $I_{d\times d}$ is the unit matrix in $\mathbb {R}^{d \times d}$ and

$$\begin{aligned} ~\Delta W_n^0=\Delta _n=t_{n+1}-t_n, ~~~~\Delta W_n^j=W_j(t_{n+1})-W_j(t_n) \end{aligned}$$

($j=1,2$) along any partitions

$$\begin{aligned} 0=t_0<t_1<t_2<.....<t_n<t_{n+1}<....<t_{n_T}=T \end{aligned}$$

of finite time intervals [0, *T*]. These methods are discretizations of *d*-dimensional stochastic differential equations (SDEs)

6 $$\begin{aligned} dX(t)=a(X(t),t) ~dt ~+~ \sum _{j=1}^m b^j(X(t),t)~dW^j(t) \end{aligned}$$

with adapted initial values $X(0)=x_0 \in \mathbb {R}^d$, where $W_j$’s are independent Wiener processes on the complete probability space $(\Omega ,\mathcal {F},\{\mathcal {F}_t\}_{t \ge 0},\mathbb {P})$ and $a, b^j: \mathbb {R}^d \times [0,T] \rightarrow \mathbb {R}^d$ are continuous vector fields. The parameter matrices $\{\Theta _n\}_{n \in \mathbb {N}} \in \mathbb {R}^{d \times d}$ determine the degree of implicitness of numerical methods (5). Without balanced terms $c^j$ and with the choice $\Theta _n=I_{d\times d}$, the scheme (5) reduces to the backward Euler-Maruyama method (BEM) and, with all $\Theta _n=0$, to the forward Euler-Maruyama method (FEM). A careful choice of matrices $c^j$ controls the path-wise and moment behavior of associated numerical solutions in a non-trivial manner (cf. Schurz (1997)).

Throughout this paper, $\Vert .\Vert =\Vert .\Vert _d$ denotes the Euclidean norm of $\mathbb {R}^d$ and $\Vert .\Vert _F$ the Frobenius norm of $\mathbb {R}^{d \times d}$. This section states the main concepts of numerical approximation of stochastic processes as it appears in an axiomatic and transparent fashion as in Schurz (2006). Let $\left( \Omega ,\mathcal {F},\{\mathcal {F}_t\}_{t \ge 0},\mathbb {P}\right)$ be a filtered, complete probability space and *Z*(*t*) be a random variable in $L^2(\Omega ,\mathcal {F}_t,\mathbb {P})$, which represents the space of square integrable random variables on the probability space $(\Omega ,\mathcal {F}_t,\mathbb {P})$ (see Shiryaev (1996) for notations and details). Moreover, $X_{Z(s),s}(t)$, $Y_{Z(s),s}(t)$ denote the one-step representations of stochastic processes *X*, *Y* evaluated at time $t\ge s$, started from $Z(s)\in L^2(\Omega ,\mathcal {F}_s,\mathbb {P})$. They are supposed to be constructable along any $\{\mathcal {F}_t\}_{t\ge 0}$-adapted discretization of the given, deterministic, finite time interval [0, *T*] and could depend on a certain mesh size $\displaystyle \Delta =\Delta _{max}=\max _{n=0,1,\ldots ,n_{T}-1}|t_{n+1}-t_n|$. Assume that there are deterministic real constants $r_0,r_2,r_{sm}\ge 0, ~0<\delta _0\le 1$ such that we have

1. **Invariance** of the analytic solution X and numerical solution Y **with respect to** $\mathbb {D} \subset \mathbb {R}^d$ (a.s. $\widehat{=}$ almost surely): That means that
  $$ \forall 0 \le t \le T\!: \mathbb {P}(X(t) \in \mathbb {D}) = 1 \quad \hbox {and} \quad \forall n \in \mathbb {N}\!: \mathbb {P}(Y_n \in \mathbb {D}) = 1. $$
2. **V-stability:** Let $V=V(Y(t))$ be a $\mathcal {F}_t$-adapted real-valued functional with $V:L^2\left( \Omega ,\mathcal {F},\{\mathcal {F}_t\}_{t \ge 0},\mathbb {P}\right) \rightarrow \mathbb {R}_+$. The numerical solution Y is V-stable, i.e., there exist a real constant $K_1$ such that
  7 $$\begin{aligned} \mathbb {E}\big [V(Y_{Y(t),t})(t+\Delta )\big |\mathcal {F}_t\big ] \le e^{2 K_1 \Delta } V(Y(t)) \end{aligned}$$
  for all t and $\Delta$ such that $0\le \Delta \le \delta _0$ and $0\le t, ~t+\Delta \le T$.
3. **Mean square contractivity of analytic solution**, i.e., there exists a real constant $K_2$ such that, for all $X(t),Y(t)\in L^2\left( \Omega ,\mathcal {F}_t,\mathbb {P}\right)$,
  8 $$\begin{aligned} \mathbb {E} \Big [\big \Vert X_{X(t),t}(t+\Delta ) - X_{Y(t),t}(t+\Delta ) \big \Vert ^2~\Big |\mathcal {F}_t\Big ] \le e^{2 K_2 \Delta }\big \Vert X(t)-Y(t)\big \Vert ^2\end{aligned}$$
  for all t and $\Delta$ such that $0\le \Delta \le \delta _0$ and $0\le t, ~t+\Delta \le T$.
4. **Mean Consistency** with rate $r_0>0$, i.e., there exists a real constant $K_3$ such that, for all $Z(t)\in L^2\left( \Omega ,\mathcal {F}_t,\mathbb {P}\right)$,
  9 $$\begin{aligned} \Big \Vert \mathbb {E} \big [X_{Z(t),t}(t+\Delta )\big | { \mathcal {F}_t } \big ] - \mathbb {E}\big [Y_{Z(t),t}(t+\Delta )\big |\mathcal {F}_t\big ]\Big \Vert \le K_3 \sqrt{V(Z(t))}~\Delta ^{r_0}\end{aligned}$$
  for all t and $\Delta$ such that $0\le \Delta \le \delta _0$ and $0\le t, ~t+\Delta \le T$, where *V* is the same functional that is used in the axiom (A2).
5. **Mean Square Consistency** with rate $r_2\!>\!0$, i.e., there exists a real constant $K_4$ such that, for all $Z(t)\in L^2\left( \Omega ,\mathcal {F}_t,\mathbb {P}\right)$,
  10 $$\begin{aligned} \Big (\mathbb {E} \Big [\big \Vert X_{Z(t),t}(t+\Delta ) - Y_{Z(t),t}(t+\Delta ) \big \Vert ^2~\big |\mathcal {F}_t\Big ]\Big )^{1/2} \le K_4 \sqrt{V(Z(t))}~\Delta ^{r_2}\end{aligned}$$
  for all t and $\Delta$ such that $0\le \Delta \le \delta _0$ and $0\le t, ~t+\Delta \le T$, where *V* is the same functional that is used in axiom (A2).
6. **Mean Square Hölder-Smoothness of Martingale Part **$M\!\!=\!\!(\!M\!(t)\!)_{0 \!\le \! t\!\le \! T}$ with rate $r_{sm} \in [0,0.5]$, i.e., there exists a real constant $K_5\ge 0$ such that, for all $X(t),Y(t)\in L^2\left( \Omega ,\mathcal {F}_t,\mathbb {P}\right)$,
  11 $$\begin{aligned} \mathbb {E} \Big [\big \Vert M_{X(t),t}(t+\Delta )-M_{Y(t),t}(t+\Delta )\big \Vert ^2 \big | \mathcal {F}_t\Big ] \le K_5~\mathbb {E} \Big [\big \Vert X(t)-Y(t)\big \Vert ^2\Big ]\Delta ^{2r_{sm}}\end{aligned}$$
  for all t and $\Delta$ such that $0\le \Delta \le \delta _0$ and $0\le t, ~t+\Delta \le T$, where
  $$M_{z,t}(t+\Delta )=X_{z,t}(t+\Delta ) -\mathbb {E}\big [X_{z,t}(t+\Delta )~\big |\mathcal {F}_t\big ]$$
  is the martingale part of *X* given $z=X(t),Y(t)$.
7. **Interplay between consistency rates** given by $r_0\ge r_2+r_{sm}\ge 1$.
8. **Initial moment boundedness**, i.e. $\mathbb {E}\big [V\!(X_0)\big ]\!\!+\!\mathbb {E}\big [V\!(Y_0)\big ] \!<\! \infty$ with *V* is as in (A2).

Stochastic approximation problems satisfying the assumptions (A1)-(A8) on $L^2\left( \Omega ,\mathcal {F},(\mathcal {F}_t)_{t \ge 0},\mathbb {P}\right)$ are also called **well-posed**. For a very transparent, axiomatic decomposition of our numerical analysis below, we shall apply the following approximation theorem based on these axioms.

### Theorem 1

(**General **$L^2$**aligned-Convergence Theorem**) (Schurz 2006) Assume that the axioms (A1)-(A8) are satisfied and, additionally, there exist a real constant $K_{initial}$ such that

$$\mathbb {E}\Big [\Vert X_0-Y_0\Vert ^2\Big ] \;<\; K_{initial} \cdot \Delta _{max}^{r}.$$

Then, the stochastic processes $X,Y \in L^2\left( \Omega ,\mathcal {F},\{\mathcal {F}_t\},\mathbb {P}\right)$ converge to each other with respect to the naturally induced $L^2$-metric

$$\begin{aligned} m(X,Y)=\Big (\big <X-Y,X-Y\big >_{L^2}\Big )^{1/2} \end{aligned}$$

with (global) convergence rate $r_g \ge r_2 + r_{sm} - 1$.

### Remark 1

Recall that the naturally induced metric of $L^2$ is given by

$$ m(X,Y) = \left( \sup _{0 \le t \le T} \int _{\omega \in \Omega } \!\!\!\!\!\!\!\langle X(t)-Y(t),X(t)-Y(t) \rangle _d \; d \mathbb {P} (\omega ) \!\right) ^{1/2} $$

along the Euclidean scalar product $\langle .,.\rangle _d$ of $\mathbb {R}^d$.

## Numerical Analysis of Stochastic SIR Model with Disease Deaths

Consider the balanced implicit methods

12 $$\begin{aligned} Y_{n+1}=Y_n + f(Y_n)\Delta _n +\sum _{j=1}^2 g^j(Y_n) \Delta W_n^j + c(Y_n) (Y_n-Y_{n+1}) \end{aligned}$$

where $\displaystyle c(Y_n)= A_n \cdot I_{3\times 3}$ with the unit matrix $I_{3\times 3}$ of $\mathbb {R}^{3\times 3}$ and

13 $$\begin{aligned} A_n:=A(Y_n)=(\alpha \!+\!\gamma \!+\!\mu \!+\!\beta I_n)~\Delta _n\!+\! K~|F_1(Y_n)~\Delta W_n^1| \!+\! \frac{K}{R_n}~|F_2(Y_n)~\Delta W_n^2| \end{aligned}$$

for a discretization of the stochastic SIR model with disease deaths and variable diffusion rates

14 $$\begin{aligned} \displaystyle dX(t)=f(X(t))~dt + g(X(t))~dW(t)\end{aligned}$$

$$\begin{aligned} \begin{aligned} \hbox {where} \quad \displaystyle X(t) =&\begin{pmatrix} S(t)\\ I(t)\\ R(t)\end{pmatrix}\text {,~~~} f(X(t))=\begin{pmatrix} -\beta S(t)I(t)+\mu (K-S(t)) \\ \beta S(t)I(t)-(\alpha +\gamma +\mu )I(t)\\ \alpha I(t)-\mu R(t) \end{pmatrix}, \\ g(X(t))=&\begin{pmatrix} -S(t)I(t)F_1(X(t)) & 0 \\ S(t)I(t)F_1(X(t)) & -I(t) F_2(X(t))\\ 0 & I(t) F_2(X(t))\end{pmatrix} \text {~~~and~~~} dW(t)=\begin{pmatrix} dW_1(t) \\ dW_2(t) \end{pmatrix}. \end{aligned} \end{aligned}$$

Recall that, the parameters $\alpha$, $\beta$, $\gamma$, $\mu$, *K* are positive and non-random, and all diffusion rate functions $F_j: \mathbb {R}^3 \rightarrow \mathbb {R}^1$ are locally Lipschitz-continuous with local Lipschitz-constants $\widetilde{L}_i$ on the bounded domain (more precisely, a prism in $\mathbb {R}^3$)

$$\begin{aligned} \mathbb {D}=\Big \{(S,I,R) \in \mathbb {R}^3\!: S>0,~I\ge 0, ~R>0,~ S+I+R \le K\Big \} \end{aligned}$$

for $i =1,2$, respectively. The stochastic processes $W^j$ are supposed to be standard Wiener processes defined on a complete probability space $(\Omega ,\mathcal {F},\{\mathcal {F}_t\}_{t\ge 0},\mathbb {P})$ and are independent of the initial value $X(0)=X_0 \in \mathbb {R}^3$ with $\mathbb {E}[\Vert X_0\Vert _3^2] < \infty$. The functions *f* and *g* are locally Lipschitz-continuous and satisfy a linear growth condition on $\mathbb {D}$. In fact, on the bounded domain $\mathbb {D}$, one can show that

15 $$\begin{aligned} \begin{aligned} \displaystyle \forall \, X,Y \in \mathbb {D}\!: \quad \Vert f(X)-f(Y)\Vert _3^2 \le&\; L_1^2 \Vert X-Y\Vert _3^2, \\ \Vert g(X)-g(Y)\Vert _{F}^2 \le&\; L_2^2 \Vert X-Y\Vert _3^2, \\ \Vert f(X)\Vert _3^2 \le&\; L_3^2 \Big (1+\Vert X\Vert _3^2 \Big ),\\ \Vert g(X)\Vert _{F}^2 \le&\; L_4^2 \Big (1+\Vert X\Vert _3^2 \Big ) \end{aligned} \end{aligned}$$

$$\begin{aligned} \begin{aligned} \hbox {where} \quad L_1^2=&\;8\beta ^2 K^2 + 2(\alpha +\gamma +\mu )^2+2\alpha ^2,\\ L_2^2=&\;4 \widetilde{L}_1 K^4 + 4 \widetilde{L}_2 K^2 + \sup _{(S,I,R) \in ~ \mathbb {D}} \Big (8K^2 F_1^2(S,I,R) + 4 F_2^2(S,I,R) \Big ),\\ L_3^2=&\;\max \Big ( 4 \beta ^2 K^4+4\mu ^2 K^2,~4\mu ^2,~ 2(\alpha +\gamma +\mu )^2+2\alpha ^2\Big ), \\ L_4^2=&\;\max \Big ( \sup _{(S,I,R) \in ~\mathbb {D}} 2 K^2 F_1^2(S,I,R), \sup _{(S,I,R)\in ~ \mathbb {D}} 2 F_2^2(S,I,R)\Big ) \end{aligned} \end{aligned}$$

with local Lipschitz constants $\widetilde{L}_i$ of the diffusion rate functions $F_j$ on $\mathbb {D}$. In passing, note that all these quantities $L_i$ are finite on $\mathbb {D}$ under the aforementioned assumptions (which does not have to be the case in $\mathbb {R}^3$).

Now, in what follows, we shall verify that the system of assumptions $(A1)-(A8)$ (i.e., the system of axioms for Theorem 1) are satisfied step-by-step. In Schurz and Tosun (2015), we have already verified $\mathbb {D}$-invariance of exact solutions *X*.

### Theorem 2

($\mathbb {D}$**-Invariance of ***Y*) Assume that the initial condition $Y_0\!=\!(\!S_0,\!I_0,\!R_0\!) \!\in \! \mathbb {D}$ (a.s.) where $\mathbb {D}$ is defined by (2) is satisfied. **Then**, the numerical solution $\{Y_i\}_{i\in \mathbb {N}}$ governed by (12) with (13) is a.s. invariant w.r.t. $\mathbb {D}$ for all choices of step sizes $\{\Delta _n\}_{n\in \mathbb {N}}$.

### Proof

By rewriting the numerical method (12), we obtain that

16 $$\begin{aligned} S_{n+1}= &  S_n + \Big [-\beta S_n I_n+\mu (K-S_n)\Big ]\Delta _n -S_n I_n F_1(Y_n) \Delta W_n^1 + A_n(S_n-S_{n+1}), \nonumber \\ I_{n+1}= &  I_n + \Big [\beta S_n I_n - (\alpha +\gamma +\mu )I_n\Big ]\Delta _n +S_n I_n F_1(Y_n) \Delta W_n^1 \nonumber \\ &  \quad - I_n F_2(Y_n) \Delta W_n^2 +A_n(I_n-I_{n+1}),\nonumber \\ R_{n+1}= &  R_n + \Big [\alpha I_n- \mu R_n\Big ]\Delta _n + I_n F_2(Y_n) \Delta W_n^2 + A_n(R_n-R_{n+1}) \end{aligned}$$

where $A_n=(\alpha +\gamma +\mu +\beta I_n)~\Delta _n+ K~|F_1(Y_n)~\Delta W_n^1| + \frac{K}{R_n}~|F_2(Y_n)~\Delta W_n^2|.$ Then, an equivalent explicit representation of (16) is given by

17 $$\begin{aligned} S_{n+1}= &  S_n + \frac{1}{1+A_n} \Big ( [-\beta S_n I_n+\mu (K-S_n)]\Delta _n -S_n I_n F_1(Y_n) \Delta W_n^1 \Big ), \nonumber \\ I_{n+1}= &  I_n \!+\! \frac{1}{1\!+\!A_n} \Big ([\beta S_n I_n \!-\! (\alpha \!+\!\gamma \!+\!\mu )I_n]\Delta _n \!+\! S_n I_n F_1(Y_n) \Delta W_n^1 \nonumber \\ \!-\! I_n F_2(Y_n) \Delta W_n^2 \Big ), R_{n+1}= &  R_n + \frac{1}{1+A_n} \Big ([\alpha I_n- \mu R_n]\Delta _n + I_n F_2(Y_n) \Delta W_n^2 \Big ). \end{aligned}$$

We use induction on $n \in \mathbb {N}$. Suppose that $S_0+I_0+R_0 \le K$ and $S_n+I_n+R_n \le K$ with $S_n, R_n >0$ and $I_n \ge 0$. This implies that

18 $$\begin{aligned} \displaystyle S_{n+1}\!+\!R_{n+1}\!+\!I_{n+1}= &  S_n\!+\!I_n\!+\!R_n + \frac{\mu \Delta _n}{1+A_n}\Big (\!K\!-\!S_n\!-\!I_n\!-\!R_n\!\Big )-\frac{\gamma I_n \Delta _n }{1\!+\!A_n}\nonumber \\\le &  S_n+I_n+R_n+ \frac{\mu \Delta _n}{1+A_n}\Big (K-S_n-I_n-R_n\Big ) \nonumber \\= &  \left( 1-\frac{\mu \Delta _n}{1+A_n}\right) (S_n+I_n+R_n)+ \frac{\mu \Delta _n}{1+A_n}K \nonumber \\\le &  \left( 1-\frac{\mu \Delta _n}{1+A_n}\right) K+ \frac{\mu \Delta _n}{1+A_n}K\nonumber \\= &  K ~~~~\text {~since~~ } \frac{\mu \Delta _n}{1+A_n} \le 1 . \end{aligned}$$

Positivity of numerical solutions (12) with (13) can be verified by substituting

$$A_n=(\alpha +\gamma +\mu +\beta I_n) \Delta _n+ K~|F_1(Y_n)\Delta W_n^1| + \frac{K}{R_n} |F_2(Y_n)\Delta W_n^2|$$

into (17). Thus, we have

19 $$\begin{aligned} S_{n+1}= &  \frac{1}{1\!+\!A_n} \Big ( \!S_n\!+\!A_n S_n+[-\beta S_n I_n\!+\!\mu (K\!-\!S_n)]\Delta _n -S_n I_n F_1(Y_n) \Delta W_n^1 \!\Big ) \nonumber \\= &  \frac{1}{1+A_n} \Big ( S_n+ (\alpha +\gamma +\mu +\beta I_n) S_n \Delta _n + K S_n |F_1(Y_n)\Delta W_n^1| \nonumber \\ &  + \frac{K S_n}{R_n} |F_2(Y_n) \Delta W_n^2| + [-\beta S_n I_n+\mu (K-S_n)]\Delta _n -S_n I_n F_1(Y_n) \Delta W_n^1 \!\Big ) \nonumber \\= &  \frac{\mu K \Delta _n}{1+A_n} + \frac{S_n}{1+A_n} \Big ( 1+ (\alpha +\gamma )\Delta _n + K~|F_1(Y_n)\Delta W_n^1|\nonumber \\ &  - I_n F_1(Y_n) \Delta W_n^1 +\frac{K}{R_n}|F_2(Y_n)\Delta W_n^2| \Big ) ~~>~~ 0, \end{aligned}$$

20 $$\begin{aligned} I_{n+1}= &  \frac{I_n}{1\!+\!A_n} \Big (\!1\!+\!A_n\!+[\beta S_n - (\alpha \!+\!\gamma \!+\!\mu )]\Delta _n +S_n F_1(Y_n) \Delta W_n^1 - F_2(Y_n) \Delta W_n^2 \Big )\nonumber \\= &  \frac{I_n}{1\!+\!A_n} \Big (\!1+(\alpha \!+\!\gamma \!+\!\mu \!+\!\beta I_n)~\Delta _n+ K |F_1(Y_n)~\Delta W_n^1| + \frac{K}{R_n} |F_2(Y_n)~\Delta W_n^2|\nonumber \\ &  +[\beta S_n - (\alpha \!+\!\gamma \!+\!\mu )]\Delta _n +S_n F_1(Y_n) \Delta W_n^1 - F_2(Y_n) \Delta W_n^2 \!\Big )\nonumber \\= &  \frac{I_n}{1\!+\!A_n} \Big (\!1+\beta (I_n+S_n)~\Delta _n+ K~|F_1(Y_n)\Delta W_n^1| +S_n F_1(Y_n) \Delta W_n^1 \nonumber \\ &  - F_2(Y_n) \Delta W_n^2 + \frac{K}{R_n}|F_2(Y_n)\Delta W_n^2|\!\Big ) \;\ge \; 0 \text {~~~~since~~ } \frac{K}{R_n} \ge 1 . \end{aligned}$$

More precisely, $I_{n+1}>0$ if $I_{n}>0$. Furthermore

21 $$\begin{aligned} R_{n+1}= &  \frac{1}{1+A_n} \Big (R_n + A_n R_n + [\alpha I_n- \mu R_n]\Delta _n + I_n F_2(Y_n) \Delta W_n^2 \Big ) \nonumber \\= &  \frac{1}{1+A_n} \Big (R_n + (\alpha +\gamma +\mu +\beta I_n) R_n \Delta _n+ K |F_1(Y_n)\Delta W_n^1| R_n \nonumber \\ &  + \frac{K}{R_n} |F_2(Y_n)\Delta W_n^2| R_n + [\alpha I_n- \mu R_n]\Delta _n + I_n F_2(Y_n) \Delta W_n^2 \Big ) \nonumber \\= &  \frac{1}{1+A_n} \Big (R_n + (\alpha +\gamma +\beta I_n) R_n \Delta _n+ \alpha I_n \Delta _n \nonumber \\ &  + K |F_1(Y_n)\Delta W_n^1| R_n + K |F_2(Y_n)\Delta W_n^2| + I_n F_2(Y_n) \Delta W_n^2 \Big ) > 0. \end{aligned}$$

Therefore, $\mathbb {P}(\{Y_n=(S_n,I_n,R_n) \in \mathbb {D}\})=1$ for all $n\in \mathbb {N}$. $\square$

The following property of *V*-stability guarantees that certain functionals *V* of the numerical population vector *Y* (such as 2nd moments) remain exponentially bounded over the course of integration under discretization (a property, which is far from trivial under unbounded noisy perturbations in the discrete case). This property is biologically important since reasonable models must "produce" bounded growth of total populations as finite environmental resources always lead to natural limitations (boundedness).

### Theorem 3

(*V***-Stability of ***Y*) The numerical approximation *Y* governed by (12) with (13) is mean V-stable on $\mathbb {D}$ with $V(y)=1+\Vert y\Vert ^2$ and mean *V*-stability constant $K_1=L_3\!+\!\frac{L_3^2\!+\!L_4^2}{2}$ while using step sizes $\Delta _n \le 1$.

### Proof

We consider the stochastic differential equation (SDE)

22 $$\begin{aligned} dX(t) = f(X(t))~dt + g(X(t))~dW(t), ~~~~t\ge 0,~~ X(0)=x_0 \in \mathbb {D}\end{aligned}$$

and its discretization based on the balanced implicit method (12) with (13)

23 $$\begin{aligned} Y_{n+1} = Y_n + \frac{1}{1+A_n}~ f(Y_n) \Delta _n + \frac{1}{1+A_n}~ g(Y_n)\Delta W_n ,~~~~Y_0=x_0\end{aligned}$$

where $A_n=(\alpha +\gamma +\mu +\beta I_n) \Delta _n+ K~|F_1(Y_n)\Delta W_n^1| + \frac{K}{R_n} |F_2(Y_n)\Delta W_n^2|$ and $Y_n=(S_n,I_n,R_n)$. One-step representations of the solution *X* and the numerical approximation *Y* are

24 $$\begin{aligned} X_{x_0,s}(t)= &  x_0 + \int ^t_s f(X_{x_0,s}(u))~ du +\int ^t_s g(X_{x_0,s}(u))~ dW(u),\nonumber \\ Y_{x_0,s}(t)= &  x_0 + \frac{1}{1+A(s,t)} ~f(x_0) \int ^t_s du + \frac{1}{1+A(s,t)}~ g(x_0) \int ^t_s dW(u) \end{aligned}$$

where *A*(*s*, *t*) is the local one-step representation of $A_n$ with $x_0=(s_0,i_0,r_0) \in \mathbb {D}$, i.e.

25 $$\begin{aligned} A(s,t) = (\alpha \!+\!\gamma \!+\!\mu \!+\!\beta i_0) |s-t| + K |F_1(x_0) \big (W_{\!1}\!(t)\!-\!W_{\!1}\!(s)\big )| + \frac{K}{r_0} |F_2(x_0)\big (W_{\!2}\!(t)\!-\!W_{\!2}\!(s)\big )|, \end{aligned}$$

and $|t-s| \le 1$ for all $\displaystyle t> s \ge 0$, and $X(s)=x_0 \in \mathbb {D}$. To estimate $1+ \mathbb {E} [\Vert Y_{x_0,s}(t)\Vert ^2]$ we start with

$$\begin{aligned} 1\!+\!\Vert Y_{x_0,s}(t)\Vert ^2= &  1 \!+\! \left\| x_0 \!+\! \frac{1}{1+A(s,t)} \Big [f(x_0)~ (t-s) + g(x_0) \big (W(t)\!-\!W(s)\big )\Big ]\right\| ^2 \\= &  1 \!+\! \Vert x_0\Vert ^2 \!+\! \left\| \frac{1}{1\!+\!A(s,t)} \left[ f(x_0)~(t-s) + g(x_0) \big (W(t)-W(s)\big )\right] \right\| ^2\\ &  + 2 \Big \langle x_0, \frac{1}{1+A(s,t)}~ \left[ f(x_0)~(t-s) + g(x_0) \Big (W(t)-W(s)\Big )\right] \Big \rangle _{\mathbb {R}^3} \\\le &  1 \!+\! \Vert x_0\Vert ^2 \!+\! \left| \frac{1}{1\!+\!A(s,t)}\right| ^2 \left\| f(x_0)~(t\!-\!s) + g(x_0) \big (W(t)\!-\!W(s)\big ) \right\| ^2 \\ &  + 2 \Big \langle x_0, \frac{1}{1\!+\!A(s,t)}~ \left[ f(x_0)~(t-s) + g(x_0) \big (W(t)-W(s)\big )\right] \Big \rangle _{\mathbb {R}^3}. \end{aligned}$$

Now, we expand the square of the last term in the first line of the inequality and also use $\left| \frac{1}{1\!+\!A(s,t)}\right| ^2 < 1$. So, one finds the estimate

$$\begin{aligned} 1\!+\!\Vert Y_{x_0,s}(t)\Vert ^2< &  1 \!+\! \Vert x_0\Vert ^2 \!+\! \left\| f(x_0)(t\!-\!s)\right\| ^2 + \left\| g(x_0) \big (W(t)\!-\!W(s)\big ) \right\| ^2 \\ &  + 2 \Big \langle f(x_0)~(t-s),~ g(x_0) \big (W(t)-W(s)\big ) \Big \rangle _{\mathbb {R}^3} \\ &  + 2 \Big \langle x_0, ~\frac{1}{1\!+\!A(s,t)}\left[ f(x_0)(t-s) + g(x_0) \big (W(t)-W(s)\big )\right] \Big \rangle _{\mathbb {R}^3}. \end{aligned}$$

By taking the expectation and using the property of Wiener process (especially, the fact that $W(t)-W(s)\sim \mathcal {N}(0,t-s)$), for all non-random $x_0$, we obtain that

$$\begin{aligned} 1\!+\! \mathbb {E} [\Vert Y_{x_0,s}(t)\Vert ^2]\le &  1 \!+\! \Vert x_0\Vert ^2 \!+\! \left\| \!f(x_0)\!\right\| ^2 (t\!-\!s)^2+ \left\| \!g(x_0) \!\right\| _F^2 \mathbb {E} \Big [\big \Vert W(t)\!-\!W(s)\big \Vert _2^2\!\Big ] \\ &  + 2 \, \mathbb {E} \Big [\Big \Vert \!x_0 \frac{1}{1\!+\!A(s,t)} f(x_0) (t\!-\!s) \Big \Vert \Big ] \\= &  1 \!+\!\Vert x_0\Vert ^2 \!+\! \left\| \!f(x_0)\!\right\| ^2 (t\!-\!s)^2+ 2\left\| \!g(x_0) \!\right\| _F^2 |t\!-\!s|^2\!\Big ] \\ &  + 2 \, \mathbb {E} \Big [\Big \Vert \!x_0 \frac{1}{1\!+\!A(s,t)} f(x_0) (t\!-\!s) \Big \Vert \Big ] \\\le &  1 \!+\! \Vert x_0\Vert ^2 \!+\! L_3^2 (1 \!+\! \Vert x_0\Vert ^2 ) (t\!-\!s)^2\!+\! L_4^2 (1 \!+\! \Vert x_0\Vert ^2 ) (t\!-\!s) \\ &  + 2 \Vert x_0\Vert \, \mathbb {E} \left[ \left| \frac{1}{1+A(s,t)}\right| \right] \Vert f(x_0)\Vert (t\!-\!s). \end{aligned}$$

Recall $A(s,t)\ge 0$ (everywhere). Since $(t-s)^2\le t-s <1$ and $\Vert x_0\Vert \le \sqrt{1+\Vert x_0\Vert ^2}$, we arrive at

$$\begin{aligned} 1+ \mathbb {E} [\Vert Y_{x_0,s}(t)\Vert ^2]\le &  \Big (\!1\!+(2L_3\!+\!L_3^2\!+\!L_4^2)(t\!-\!s)\!\Big ) (1 \!+\! \Vert x_0\Vert ^2 ) \\\le &  e^{(2L_3\!+\!L_3^2\!+\!L_4^2)(t\!-\!s)} (1 \!+\! \Vert x_0\Vert ^2). \end{aligned}$$

The last step of our estimation procedure is obtained by the Taylor series expansion of exponentials at *s*, i.e., we use the elementary inequality $e^{a(t-s)} \ge 1+ a(t-s)$ for $a\ge 0, t\ge s$. Hence, the numerical approximation *Y* governed by (12) with (13) and step sizes $\Delta _n \le 1$ is V-stable with $V(x_0)=1+\Vert x_0\Vert ^2$ and *V*-stability constant $K_1=L_3\!+\!\frac{L_3^2\!+\!L_4^2}{2}$. $\square$

In passing, we note that the notion of mean square contractivity is introduced by Schurz (1997). Mean square contractivity means that neighbored biologic populations with different initial data stay neighbored through their evolution and distances between them can be controlled exponentially both in space and in time.

### Theorem 4

(**Mean Square Contractivity of ***X*) *X* is mean-square contractive with constant $K_2 \le 2|L_1|+L_2^2$ of contractivity on $\mathbb {D}$.

### Proof

We shall follow similar ideas and concepts as in Schurz (2001a), Schurz (2001b) on contractivity of moment-dissipative stochastic systems. Consider the 6-dimensional system (i.e., of 2 identical copies of the same 3-dimensional SDEs for *X*, but started at $x\ne y$ at time *s*)

$$\begin{aligned} dX_1(t)&=f(X_1(t)) dt + g(X_1(t)) dW(t), \quad t \ge s, \quad X_1(s)=x,\\ dX_2(t)&=f(X_2(t)) dt + g(X_2(t)) dW(t), \quad t \ge s, \quad X_2(s)=y. \end{aligned}$$

Now, calculate the associated infinitesimal generator $\mathcal {L}$ applied to the positive functional $\Vert x-y\Vert ^2$. Then, by Dynkin’s formula Dynkin (1965), we obtain that

$$\begin{aligned} &  \mathbb {E}\Big [\!\Vert X_{\!x,s}\!(t)\!-\!X_{\!y,s}\!(t)\Vert ^2\!\Big ]\\ &  \quad =\mathbb {E} [\Vert x\!-\!y\Vert ^2] \!+\! 2 \mathbb {E} \Big [\!\!\int ^t_s\!\! \!\!\Big \langle \!\!X_{\!x,s}\!(\!u\!)\!-\!\!X_{\!y,s}\!(\!u\!),\!f(\!X_{\!x,s}\!(\!u\!)\!)\! -\!\!f(\!X_{\!y,s}\!(\!u\!)\!)\!\!\Big \rangle _{\!\mathbb {R}^3} \!\!\!+\!\!\Big \Vert g(\!X_{\!x,s}\!(\!u\!)\!)\!-\!\!g(\!X_{\!y,s}\!(\!u\!)\!)\!\Big \Vert ^2 \!\!du\!\Big ] \\ &  \quad \le \Vert x\!-\!y\Vert ^2\!+\!\big (2 |L_1|\!+\!L_2^2\big )\!\!\int ^t_s\!\!\mathbb {E} \Big [\!\Vert X_{\!x,s}\!(u)\!-\!X_{\!y,s}\!(u)\Vert ^2\Big ] du. \end{aligned}$$

Next, we apply the well-known Gronwall inequality in order to arrive at

26 $$\begin{aligned} \displaystyle \mathbb {E}\left[ \left\| X_{\!x,s}\!(t)\!-\!X_{\!y,s}\!(t)\right\| ^2\right] \,\le \, \Vert x-y\Vert ^2~e^{\big (2 L_1^2\!+\!L_2^2\big )(t-s)}. \end{aligned}$$

Finally, we may exploit standard properties of conditional expectations (such as the tower property) and, for all $X\!(t),Y\!(t) \in L^2(\Omega ,\mathcal {F}_t,\mathbb {P})$, we find that

$$\begin{aligned} \mathbb {E}\Big [\Big \Vert X_{\!X\!(t),t}\!(t\!+\!\Delta )\!-\!X_{\!Y\!(t),t} \!(t\!+\!\Delta )\Big \Vert ^2\Big |\mathcal {F}_t\Big ] \,\le \, \Vert X\!(t)-Y\!(t)\Vert ^2~e^{\big (2 L_1^2\!+\!L_2^2\big )\Delta } \end{aligned}$$

with any nonrandom $\Delta >0$. This proves the mean square contractivity of exact solutions *X* with constant $K_2 \le 2|L_1|+L_2^2$ of contractivity for all $x,y \in \mathbb {D}$ (as *X* stays on $\mathbb {D}$ (a.s.)). $\square$

Both, the exact and numerical solutions have mean square bounded evolutions of total populations, which can be controlled by the initial data. That is the content of the following theorem.

### Theorem 5

(**Local Mean Square Boundedness of ***X*, *Y*** By Initial Data**) Let $\Delta t = t-s \le 1$ for *Y*. Then, both the analytic solution *X* and the numerical approximation *Y* governed by (12) with (13) are mean-square bounded on $\mathbb {D}$.

### Proof

By the help of the Hölder’s inequality

$$\begin{aligned} \displaystyle \left\| \int ^t_s B(u)~du\right\| ^2 \le \left( \int ^t_s \Vert 1\Vert ^2 ~du\right) \left( \int ^t_s \Vert B(u)\Vert ^2 ~du\right) , \end{aligned}$$

the elementary inequality $(a+b+c)^2 \le 3(a^2+b^2+c^2)$ and the Itô isometry relation

$$\displaystyle \mathbb {E} \Big [\!\left\| \!\int ^t_s \!\!B(u)~ dW(u)\right\| ^2\Big ] = \mathbb {E} \Big [\!\int ^t_s \left\| B(u)\right\| ^2~ du\Big ]$$

with adapted integrands *B*, we obtain that

$$\begin{aligned} \begin{aligned} 1\!+\!\mathbb {E} [\Vert&X_{x_0,s}(t)\Vert ^2] \\=&\, 1\!+\! \mathbb {E}\Big [\left\| \!x_0\!+\!\! \int ^t_s \!\!f(X_{x_0,s}(u)) du \!+\!\! \int ^t_s \!\!g(X_{x_0,s}(u)) dW(u) \!\right\| ^2\Big ] \\ \le&\, 1\!+\! 3\Vert x_0\Vert ^2\!+\! 3\mathbb {E} \Big [\!\left\| \!\int ^t_s \!\!f(X_{x_0,s}(u)) du \!\right\| ^2\!\Big ]\!+\! 3\mathbb {E} \Big [\left\| \!\int ^t_s \!\!g(X_{x_0,s}(u)) dW(u) \!\right\| ^2\Big ] \\ \le&\, 1\!+\! 3\Vert x_0\Vert ^2\!+\! 3 (t-s) \mathbb {E} \Big [\!\int ^t_s \!\!\Vert f(X_{x_0,s}(u))\Vert ^2 du\Big ] \!+\! 3\mathbb {E} \Big [\!\int ^t_s \!\!\Vert g(X_{x_0,s}(u)) \Vert _F^2 \, du\Big ].\\ \end{aligned} \end{aligned}$$

Now, we use local Lipschitz-continuity of *f* and *g* (see (15)). Then, the latter inequality becomes

27 $$\begin{aligned} \begin{aligned} 1+\mathbb {E} [\Vert X_{x_0,s}(t)\Vert ^2] \le&\; 1+ 3\Vert x_0\Vert ^2+ 3 L_3^2~\int ^t_s \Big (1+\mathbb {E} \Big [\Vert X_{x_0,s}(u)\Vert ^2\Big ]\Big ) du\\&\quad + 3 L_4^2~\int ^t_s \Big (1+\mathbb {E} \Big [\Vert X_{x_0,s}(u)\Vert ^2\Big ]\Big ) du \text {~~~~~~by~~} t-s < 1 \\ \le&\; 3+ 3\Vert x_0\Vert ^2+ 3 (L_3^2+L_4^2)~\int ^t_s \Big (1+\mathbb {E} \Big [\Vert X_{x_0,s}(u)\Vert ^2\Big ]\Big ) du \\ \le&\; 3\Big (1+ \Vert x_0\Vert ^2\Big )~e^{3 (L_3^2+L_4^2)(t-s)} \;\le \; c_1^2 \Big (1+ \Vert x_0\Vert ^2\Big ) \end{aligned} \end{aligned}$$

by standard Gronwall inequality, where $c_1^2 = 3 e^{3 (L_3^2+L_4^2)}$. This proves the local uniform boundedness of *X*. Similarly, we find that

$$\begin{aligned} \displaystyle 1+ &  \Vert Y_{x_{0},s}(t)\Vert ^2 \\= &  \, 1+ \left\| x_0+\frac{1}{1+A(s,t)} f(x_0)\int ^t_s ~du + \frac{1}{1+A(s,t)} g(x_0)\int ^t_s ~dW(u)\right\| ^2 \\\le &  \, 1+ 3\Vert x_0\Vert ^2+ 3 \left| \frac{1}{1+A(s,t)}\right| ^2 \Vert f(x_0)\Vert ^2 \left| \int ^t_s \!du\right| ^2 \\ &  + 3 \left| \frac{1}{1+A(s,t)}\right| ^2 \Vert g(x_0)\Vert _F^2 \left\| \int ^t_s \!dW(u)\right\| ^2 \\\le &  \, 1+ 3\Vert x_0\Vert ^2+ 3 L_3^2(1+\Vert x_0\Vert ^2)(t-s)^2 + 3 L_4^2(1+\Vert x_0\Vert ^2) \left\| \int ^t_s dW(u)\right\| ^2 \end{aligned}$$

where *A*(*s*, *t*) is of the form (25). By taking the expectation we obtain

$$\begin{aligned} \displaystyle 1+ &  \mathbb {E} [\Vert Y_{x_{0},s}(t)\Vert ^2] \\\le &  \, 1+ 3\Vert x_0\Vert ^2+ 3 L_3^2(1+\Vert x_0\Vert ^2)(t-s)^2 + 3 L_4^2(1+\Vert x_0\Vert ^2) ~\mathbb {E}~\Big [\left\| \!\int ^t_s dW(u)\!\right\| ^2\Big ] \\= &  \, 1+ 3\Vert x_0\Vert ^2+ 3 L_3^2(1+\Vert x_0\Vert ^2)(t-s)^2 + 3 L_4^2(1+\Vert x_0\Vert ^2) ~\mathbb {E} \Big [\!\int ^t_s \!\Vert 1\Vert ^2 du\Big ] \\\le &  \, 3+ 3\Vert x_0\Vert ^2+ 3 L_3^2(1+\Vert x_0\Vert ^2)(t-s)^2 + 3 L_4^2(1+\Vert x_0\Vert ^2) (t-s)\\\le &  \, 3\Big [1+(L_3^2+L_4^2)(t-s)\Big ] \Big (1+\Vert x_0\Vert ^2\Big ) \le c_2^2 \Big (1+\Vert x_0\Vert ^2\Big ) \end{aligned}$$

where $c_2^2 = 3\Big [1+(L_3^2+L_4^2)\Big ]$. That completes the proof. $\square$

Hölder continuity with Hölder exponents measures how smooth the solutions of our models are expected to be. It turns out that the larger the Hölder exponent the smoother the solutions are. Under random perturbations, we can establish mean square Hölder continuity with Hölder exponents 0.5 as follows.

### Theorem 6

(**Mean Square Hölder Continuity of ***X*, *Y*) Both, the exact solution *X* and the continuous time representation of numerical approximation *Y* are locally mean-square Hölder continuous on $\mathbb {D}$ with Hölder exponent 0.5.

### Proof

We begin with the local mean square continuity of the exact solution *X*.

$$\begin{aligned}\begin{aligned} \mathbb {E}\Big [\Vert X_{x_0,s}&(t)-x_0\Vert ^2\Big ]\\ =&\, \mathbb {E}\Big [\left\| \!\int ^t_s\!\! f(X_{x_0,s}(u))~du + \!\int ^t_s \!\!g(X_{x_0,s}(u))~dW(u)\!\right\| ^2\Big ] \\ \le&\, 2\mathbb {E}\Big [\left\| \!\int ^t_s \!\!f(X_{x_0,s}(u))~du \!\right\| ^2\Big ]+ 2\mathbb {E} \Big [\left\| \!\int ^t_s \!\!g(X_{x_0,s}(u))~dW(u)\!\right\| ^2\Big ] \\ \le&\, 2 (t-s)\mathbb {E}\Big [\!\int ^t_s \!\!\Vert f(X_{x_0,s}(u)) \Vert ^2~du\Big ]+ 2 \mathbb {E}\Big [\!\int ^t_s \!\!\Vert g(X_{x_0,s}(u)) \Vert _{F}^2~du\Big ] \\ \le&\, 2 (L_3^2\!+\!L_4^2)\!\int ^t_s \!\!\Big (1+\mathbb {E}\Big [\Vert X_{x_0,s}(u) \Vert ^2\Big ]\Big )~du. \end{aligned} \end{aligned}$$

By the local uniform boundedness (27) of *X*, we obtain that

28 $$\begin{aligned} \mathbb {E}\Big [\Vert X_{x_0,s}(t)-x_0\Vert ^2\Big ]\le &  2 (L_3^2+L_4^2)(1\!+\!\Vert x_0 \Vert ^2)\!\int ^t_s \!\!3 e^{3 (L_3^2\!+\!L_4^2)(u\!-\!s)}~du \nonumber \\= &  6 (L_3^2+L_4^2)\Big (1+\Vert x_0 \Vert ^2\Big )\frac{e^{3 (L_3^2+L_4^2)(t-s)}-1}{3 (L_3^2+L_4^2)}\nonumber \\= &  2 \Big [e^{3 (L_3^2+L_4^2)(t-s)}-1\Big ]\Big (1+\Vert x_0 \Vert ^2\Big )\nonumber \\= &  2 \frac{e^{3 (L_3^2+L_4^2)(t-s)}-1}{t-s} \Big (1+\Vert x_0 \Vert ^2\Big )(t-s).\end{aligned}$$

We may choose a finite positive constant $c_3$ by

$$2 \max _{|z|\le 1} \frac{e^{3 (L_3^2+L_4^2)z}-1}{z} =: c_3^2 <+\infty .$$

Hence, for non-random $x_0$, we have

29 $$\begin{aligned} \displaystyle \mathbb {E}\Big [\Vert X_{x_0,s}(t)-x_0\Vert ^2\Big ] \le c_3^2(1+\Vert x_0 \Vert ^2)(t-s). \end{aligned}$$

This proves the local Hölder continuity of *X* in the mean square sense (with exponent 1/2 in time and local mean square Hölder constant $c_3^2$).

Now, we work on the local Hölder-continuity of the continuous time implementation of numerical approximation *Y* governed by BIMs (12) with (13). We find that

$$\begin{aligned} \Vert Y_{x_0,s}(t)-x_0\Vert ^2 = \left\| \frac{1}{1+A(s,t)} f(x_0)\int ^t_s\!du + \frac{1}{1+A(s,t)} g(x_0)\int ^t_s\!\!dW(u)\right\| ^2 \end{aligned}$$

where *A*(*s*, *t*) satisfies (25). We encounter with the estimation

$$\begin{aligned} \Vert Y_{x_0,s}(t)-x_0\Vert ^2\le &  \left| \frac{1}{1+A(s,t)}\right| ^2 \Vert f(x_0)\Vert ^2 \left| \int ^t_s\!du\right| ^2 \\  &  + \left| \frac{1}{1+A(s,t)}\right| ^2 \Vert g(x_0)\Vert _{F}^2 \left\| \int ^t_s\!\!dW(u)\right\| ^2 \\ &  +2 \Big<\!\frac{1}{1+A(s,t)} f(x_0)\!\int ^t_s \!du, \!\frac{1}{1+A(s,t)} g(x_0)\!\int ^t_s\!\!dW(u) \!\Big>_{\mathbb {R}^3}\\\le &  L_3^2 (1+\Vert x_0\Vert ^2)(t\!-\!s)^2+ L_4^2 (1+\Vert x_0\Vert ^2) \left\| W(t)-W(s)\right\| ^2\\ &  {+} 2 \Big <\!\frac{1}{1{+}A(s,t)} f(x_0) [t-s], \frac{1}{1{+}A(s,t)} g(x_0)[W(t)-W(s)]\!\Big >_{\mathbb {R}^3}. \end{aligned}$$

Next, we take the expectation and apply Lemma 1 from the appendix to get to

30 $$\begin{aligned} \mathbb {E}\Big [\!\Vert Y_{x_0,s}(t)\!-\!x_0\Vert ^2\!\Big ]\le &  L_3^2 (1\!+\!\Vert x_0\Vert ^2)(t-s)^2+ L_4^2 (1\!+\!\Vert x_0\Vert ^2) \mathbb {E}\Big [\!\left\| W(t)-W(s)\right\| ^2\!\Big ] \nonumber \\ &  +2 \underbrace{\mathbb {E}\Big [\Big \langle \frac{f(x_0)}{1+A(s,t)} [t-s], \frac{1}{1+A(s,t)} g(x_0) [W(t)-W(s)] \Big \rangle _{\mathbb {R}^3}\Big ]}_{\displaystyle = 0 \quad \hbox {by Lemma }1 }\nonumber \\\le &  L_3^2 (1+\Vert x_0\Vert ^2)(t-s)^2+ 2 L_4^2 (1+\Vert x_0\Vert ^2) (t-s) \nonumber \\\le &  (L_3^2+2L_4^2)(t-s) (1+\Vert x_0\Vert ^2) \nonumber \\\le &  c_4^2(t-s) (1+\Vert x_0\Vert ^2) \end{aligned}$$

Hence, the proof is complete. $\square$

Next, we verify the mean and mean square consistency of our approximations (12). Consistency guarantees us that the underlying vector field components of our models are indeed locally approximated. We shall also estimate local rates of consistency. That also means that the relevant biologic populations are replicated well in a local sense.

### Theorem 7

(**Mean Consistency of ***Y*) Assume that $\displaystyle \frac{|F_2(S,I,R)|}{R}$ and $\displaystyle |F_1(S,I,R)|$ are bounded functions on $\mathbb {D}$, i.e.

31 $$\begin{aligned} \sup _{(S,I,R) \in \mathbb {D}} \displaystyle \frac{|F_2(S,I,R)|}{R} + \sup _{(S,I,R) \in \mathbb {D}} |F_1(S,I,R)| \;<\; +\infty . \end{aligned}$$

Then, the numerical approximation *Y* governed by BIMs (12) with weights (13) is mean consistent on $\mathbb {D}$ with local rate $r_0 \!\ge \! 1.5$ and leading constant $K_3\!=\!\frac{2}{3}L_1\!c_3 + (a\!+\!b) L_3$ of mean consistency where $a=\alpha \!+\! \gamma \!+\! \mu \!+\! \beta K$, *b* is defined by (33), and

$$ c_3^2 = 2 \max _{|z|\le 1} \frac{e^{3 (L_3^2+L_4^2)z}-1}{z} < +\infty . $$

### Proof

We start with the estimation of $\mathbb {E}\left[ \frac{A(s,t)}{1\!+\!A(s,t)}\right]$ and $\mathbb {E}\left[ \frac{A(s,t)}{1\!+\!A(s,t)} \int ^t_s dW(u)\right]$ where the expression $A(s,\!t)$ with $x_0=(x_{0,1},x_{0,2},x_{0,3})\in \mathbb {D}$ is defined by

$$\begin{aligned} A(s,\!t)\!=\! &  (\alpha \!+\!\gamma \!+\!\mu \!+\!\beta x_{0,2}\!)(t\!-\!s) \!+\! K \!\left| \!F_1\!(x_0)\big (\!W_1\!(t)\!-\!W_1\!(s)\!\big )\!\right| \\  &  \quad \!+\! \frac{K}{\!\!x_{0,3}\!}\!\left| \!F_2\!(x_0)\big (\!W_2\!(t)\!-\!W_2\!(s)\!\big ) \!\right| \!. \end{aligned}$$

Because $Y_{x_0,s}(t)$ is invariant with respect to the bounded domain $\mathbb {D}$, we can estimate

32 $$\begin{aligned} A(s,t)\le &  a(t-s)+b \Vert W(t)-W(s)\Vert \end{aligned}$$

with appropriate finite constants *a* and *b* (under (31)), which are determined by

33 $$\begin{aligned} a = \alpha \!+\! \gamma \!+\! \mu \!+\! \beta K, \quad b = K \Big (\!\sup _{(S<I,R) \in \mathbb {D}} \!\!\!\!\!\!|F_1(S,I,R)| + \!\!\!\!\sup _{(S<I,R) \in \mathbb {D}} \!\!\!\!\!\!\!\!\dfrac{|F_2(S,I,R)|}{R}\Big ) < +\infty . \end{aligned}$$

Since $\displaystyle ~0< \frac{1}{1+A(s,t)}<1~$, then $\displaystyle ~0< \frac{A(s,t)}{1+A(s,t)}<A(s,t)$ for all *s*, *t*. Now, we take the expectation and obtain the inequality

$$ \displaystyle \mathbb {E}\left[ \frac{A(s,t)}{1+A(s,t)}\right] < \mathbb {E}[A(s,t)] \le a \, (t-s)+ b \, \mathbb {E}\Big [\Vert W(t)-W(s)\Vert \Big ] $$

with constants *a* and *b* satisfying (33). Next, we use the Gaussian property $\displaystyle W_j(t)-W_j(s)\sim \mathcal {N} (0,t-s)$ ($j=1,2$) in order to conclude that

34 $$\begin{aligned} \displaystyle \mathbb {E}\left[ \frac{A(s,t)}{1+A(s,t)}\right] \le a (t-s)+b (t-s)^{1/2}. \end{aligned}$$

Similarly, due to the symmetry of Gaussian distributions, the independence of $W_1$ and $W_2$, and the independence of $X(s)=(S(s),I(s),R(s))$ from the Gaussian distributed increments $W_j(t)-W_j(s)$, we obtain that

35 $$\begin{aligned}&\displaystyle \left\| \mathbb {E}\left[ \frac{A(s,t)}{1+A(s,t)} \int ^t_s \!\!dW(u)\right] \right\| ^2 = \displaystyle \left\| \mathbb {E}\left[ \frac{A(s,t)}{1+A(s,t)} [W(t)-W(s)]\right] \right\| ^2\nonumber \\ =&\displaystyle \Big (\!\underbrace{\mathbb {E}\left[ \frac{A(s,t)}{\!1\!+\!A(s,t)\!} [W_1(t)\!-\!W_1(s)]\right] }_{\displaystyle = 0}\!\Big )^{\!\!2} \!+ \displaystyle \Big (\!\underbrace{\mathbb {E}\left[ \frac{A(s,t)}{\!1\!+\!A(s,t)\!} [W_2(t)\!-\!W_2(s)]\right] }_{\displaystyle = 0}\!\Big )^{\!\!2} = 0. \end{aligned}$$

Note that the absolute values $|W_k(t)-W_k(s)|$ only enter in the scalar quotient $A(s,t)/[1\!+\!A(s,t)]$. Now, to prove the mean consistency of *Y* to *X*, we first find an explicit expression for the difference $X_{x_0,s}(t)-Y_{x_0,s}(t)$ as follows.

$$\begin{aligned} \displaystyle X_{x_0,s}(t)-Y_{x_0,s}(t)= &  \int ^t_s \!\!f(X_{x_0,s}(u)) \, du + \int ^t_s \!\!g(X_{x_0,s}(u)) \, dW(u) \\ &  -\frac{1}{1\!+\!A(s,t)} f(x_0)\int ^t_s \!\!du -\frac{1}{1+A(s,t)} g(x_0)\int ^t_s\!\!dW(u)\\= &  \int ^t_s \!\!\Big [\!f(X_{x_0,s}(u))\!-\!f(x_0)\!\Big ] du + \int ^t_s\!\!\Big [\!g(X_{x_0,s}(u))\!-\!g(x_0)\!\Big ] dW(u) \\ &  {+} \!\left( \!1\!-\!\frac{1}{\!1\!+\!A(s,t)\!}\!\right) f(x_0)\!\int ^t_s\!\!\!\!du {+} \!\left( \!1\!-\!\frac{1}{\!1{+}A(s,t)\!}\!\right) g(x_0)\!\int ^t_sdW(u). \end{aligned}$$

Next, we take the expectation, the Euclidean norm and $\triangle$-inequality. Thus

36 $$\begin{aligned}&\displaystyle \Big \Vert \mathbb {E} \Big [X_{x_0,s}(t)\!-\!Y_{x_0,s}(t)\Big ]\Big \Vert \nonumber \\&\;\,\le \left\| \mathbb {E} \Big [\!\int ^t_s \!\!\left[ f(X_{x_0,s}(u))-f(x_0)\right] du\Big ]\right\| \nonumber \\&\quad + \left\| f(x_0)(t\!-\!s)~\mathbb {E} \left[ \!\frac{A(s,t)}{1\!+\!A(s,t)}\!\right] \right\| + \Big \Vert g(x_0) ~\underbrace{\mathbb {E}\!\left[ \!\frac{A(s,t)}{1\!+\!A(s,t)} \!\int ^t_s \!\!\!dW(u)\!\right] } _{\displaystyle = (0,0)^T \; \text{ by } \; (35)}\Big \Vert \nonumber \\&\!\!\!\!\!\!{\mathop {\le }\limits ^{(34),(35)}}\! \!\left\| \mathbb {E} \!\Big [\!\int ^t_s \!\!\!\!\left[ f(X_{x_0,s}(u))\!-\!f(x_0)\right] du \Big ]\!\right\| \!+\! a \left\| f(x_0)\right\| \!(t\!-\!s)^2 \!\!+ \!b \left\| f(x_0)\right\| \!(t\!-\!s)^{3/2}\nonumber \\&\;\le \mathbb {E}\left[ \left\| \!\int ^t_s \!\!\!\!\left[ f\!(\!X_{x_0\!,s}\!(u)\!)\!-\!f\!(\!x_0\!)\right] \!du\right\| \right] \!\!+\! a L_3\sqrt{\!1\!\!+\!\!\Vert x_0\Vert ^2\!}(t\!\!-\!\!s)^2\!+\! b L_3 \sqrt{\!1\!\!+\!\!\Vert x_0\Vert ^2\!}(t\!\!-\!\!s)^{3/2}\!\!\!\!. \end{aligned}$$

The first term of the right hand side of the above inequality (36) can be estimated by

37 $$\begin{aligned} \begin{aligned} \displaystyle \mathbb {E}&\left[ \left\| \int ^t_s \!\!\left[ f(X_{x_0,s}(u))\!-\!f(x_0)\right] du\right\| \right] \le \int ^t_s \!\!\mathbb {E}\Big [\!\left\| f(X_{x_0,s}(u))\!-\!f(x_0)\right\| \!\Big ] du \\ \le&\; L_1 \!\int ^t_s \!\!\mathbb {E}\Big [\Vert X_{x_0,s}(u)\!-\!x_0\Vert \Big ] du \; \le \; L_1 \!\!\int ^t_s\!\!\!\left( \mathbb {E}\Big [\Vert X_{x_0,s}(u)\!-\!x_0\Vert ^2\Big ]\right) ^{\!\!1/2} \!\!\!du \\ \le&\; L_1 c_3 \!\int ^t_s \!\!\sqrt{1\!+\!\Vert x_0\Vert ^2} \, (u-s)^{1/2} \, du \quad \hbox {by inequality} \; (29). \end{aligned} \end{aligned}$$

Hence, after substituting (37) into (36), we obtain that

$$\begin{aligned} \displaystyle \left\| \mathbb {E} \Big [X_{x_0,s}(t)-Y_{x_0,s}(t)\Big ]\right\| \;\le \left( \!\frac{2}{3}L_1c_3 + (a+b) L_3\!\right) \sqrt{1\!+\!\Vert x_0\Vert ^2} \, (t\!-\!s)^{3/2}\!\!\!. \end{aligned}$$

Therefore, the numerical method *Y* governed by BIMs (12) with weights (13) is mean consistent with rate $\displaystyle r_0=1.5$. $\square$

### Theorem 8

(**Mean Square Consistency of ***Y*) Assume that the functions $\displaystyle \frac{|F_2(S,I,R)|}{R}$ and $\displaystyle |F_1(S,I,R)|$ are bounded functions on $\mathbb {D}$, i.e., the relation (31) holds with

38 $$\begin{aligned} \sup _{(S,I,R) \in \mathbb {D}} \displaystyle \frac{|F_2(S,I,R)|}{R} + \sup _{(S,I,R) \in \mathbb {D}} |F_1(S,I,R)| \;<\; +\infty . \end{aligned}$$

Then, the numerical method *Y* governed by BIMs (12) with weights (13) is mean-square consistent on $\mathbb {D}$ with local rate $r_2 =1.0$ and leading constant of mean-square consistency

$$\begin{aligned} K_4=\sqrt{2c_3^2(L_1^2+L_2^2)+4 L_3^2+8(a^2+3b^2)L_4^2} \end{aligned}$$

where $a, b, c_3$ are defined as in Theorem 7.

### Proof

Recall the elementary inequality $(a+b+c+d)^2\le 4(a^2+b^2+c^2+d^2)$. We start with the estimation

$$\begin{aligned} \begin{aligned} \displaystyle \mathbb {E}\Big [\Vert&X_{x_0,s}(t)\!-\!Y_{x_0,s}(t)\Vert ^2\Big ]\\ =&\; \mathbb {E} \Big [\Big \Vert \int ^t_s \!\!\left[ f(X_{x_0,s}(u))\!-\!f(x_0)\right] du + \!\int ^t_s \!\!\left[ g(X_{x_0,s}(u))\!-\!g(x_0)\right] dW(u) \\&+ \left( \!1\!-\!\frac{1}{1\!+\!A(s,t)}\!\right) f(x_0)\!\int ^t_s \!\!du + \left( 1\!-\!\frac{1}{1\!+\!A(s,t)}\right) g(x_0)\!\int ^t_s \!\!dW(u)\Big \Vert ^2 \Big ]\\ \le&\; 4\mathbb {E}\left[ \left\| \int ^t_s \!\!\!\left[ f(X_{x_0,s}(u))-f(x_0)\right] du\right\| ^2\right] \\ &\quad +4\mathbb {E}\left[ \left\| \int ^t_s \!\!\!\left[ g(X_{x_0,s}(u))-g(x_0)\right] dW(u)\right\| ^2\right] \\&\; +4\mathbb {E}\Big [\left\| \frac{A(s,t)}{1\!+\!A(s,t)} f(x_0)\!\int ^t_s\!\!du\right\| ^2\Big ] + 4\mathbb {E}\Big [\left\| \frac{A(s,t)}{1\!+\!A(s,t)} g(x_0)\!\int ^t_s\!\!dW(u)\right\| ^2\Big ] \\ \le&\; 4(t-s) \mathbb {E} \Big [\!\int ^t_s \!\!\left\| f(X_{x_0,s}(u))\!-\!f(x_0)\right\| ^2 du\Big ] + 4\mathbb {E} \Big [\!\int ^t_s \!\!\left\| g(X_{x_0,s}(u))\!-\!g(x_0)\right\| ^2 \Big ] du\\&\; + 4(t-s)^2\mathbb {E}\left[ \left\| f(x_0)\right\| ^2\right] + 4\mathbb {E}\left[ \Vert g(x_0)\Vert _F^2\left\| \frac{A(s,t)}{1\!+\!A(s,t)}\!\int ^t_s \!\!dW(u)\right\| ^2\right] . \end{aligned} \end{aligned}$$

Next, we estimate the last part of the above inequality $\displaystyle \mathbb {E}\Big [\left\| \frac{A(s,t)}{1\!+\!A(s,t)} \!\int ^t_s \!\!dW(u)\right\| ^2\Big ]$ by using basic properties of Wiener processes *W*, the Itô isometry and Cauchy-Bunyakovsky-Schwarz inequality. Thus, we arrive at

$$\begin{aligned} \frac{A(s,t)}{1\!+\!A(s,t)} \int ^t_s \!\!\!dW(u)\le &  \frac{A(s,t)}{1+A(s,t)} \left\| \int ^t_s \!\!dW(u)\right\| \le A(s,t) \left\| \int ^t_s \!\!dW(u)\right\| ,\\ \left\| \frac{A(s,t)}{1\!+\!A(s,t)} \int ^t_s \!\!\!dW(u)\right\| ^2\le &  \left| A(s,t)\right| ^2 \left\| \int ^t_s \!\!dW(u)\right\| ^2\\&{\mathop {\le }\limits ^{(32)}}&2 \Big (a^2(t\!-\!s)^2 \!+\! b^2 \Vert W(t)\!-\!W(s)\Vert ^2 \Big ) \Vert W(t)\!-\!W(s)\Vert ^2,\\ \displaystyle \mathbb {E}\Big [\!\left\| \frac{A(s,t)}{\!1\!+\!A(s,t)\!\!} \!\int ^t_s \!\!\!\!\!dW\!(u)\right\| ^2\!\Big ]\le &  \!2 a^2(t\!-\!s)^2\mathbb {E} \Big [\!\Vert W(t)\!-\!W(s)\Vert ^2\!\Big ] \!\\  &  \quad +\! 2 b^2 \mathbb {E}\Big [\!\Vert W(t)\!-\!W(s)\Vert ^4\!\Big ] \\\le &  2 a^2(t-s)^2(t-s) + 2 b^2 3(t-s)^2 \\= &  2 a^2(t-s)^3 + 6 b^2 (t-s)^2 \end{aligned}$$

where the finite constants *a* and *b* are given by (33) under (38). Therefore, we have

$$\begin{aligned} \begin{aligned} \mathbb {E}\Big [\Vert&X_{x_0,s}(t)-Y_{x_0,s}(t)\Vert ^2\Big ]\\ \,\le \;\;&4(t-s) L_1^2 \!\int ^t_s \!\!\mathbb {E}\Big [\left\| X_{x_0,s}(u)\!-\!x_0\right\| ^2 \Big ] du + 4 L_2^2 \!\int ^t_s \!\!\mathbb {E}\Big [\left\| X_{x_0,s}(u)\!-\!x_0\right\| ^2\Big ] du\\&+4(t\!-\!s)^2 L_3^2 \Big (\!1\!+\!\Vert x_0\Vert ^2\!\Big ) +4 L_4^2 \Big (\!2 a^2(t\!-\!s)^3 + 6 b^2 (t\!-\!s)^2 \!\Big )\Big (\!1\!+\!\Vert x_0\Vert ^2\!\Big )\\ \!\!\!\!{\mathop {\le }\limits ^{(29)}}&4\Big ( L_1^2(t-s)+L_2^2\Big ) \left[ \frac{c_3^2}{2} (t-s)^2 \Big (1+\Vert x_0\Vert ^2\Big )\right] \\&+4\Big [L_3^2(t-s)^2+ L_4^2 \big (2 a^2(t-s)^3 + 6 b^2 (t-s)^2 \big )\Big ] \Big (1+\Vert x_0\Vert ^2\Big )\\ \,\le \;\;&c_5^2(t-s)^2\Big (1+\Vert x_0\Vert ^2\Big ) \end{aligned} \end{aligned}$$

where $c_5^2= 2c_3^2(L_1^2+L_2^2)+4 L_3^2+8(a^2+3b^2)L_4^2$. Hence, the numerical method *Y* governed by BIMs (12) with weights (13) is mean square consistent with rate $\displaystyle r_2=1.0$ of $L^2$-convergence. $\square$

Next, we shall show that the axiom (*A*6) is also fulfilled. That means that the noisy part of the dynamics of *X* is also Hölder continuous.

### Theorem 9

(**Hölder Continuity of Martingale Part of ***X*) All solutions $X=(S,R,I)$ obeying the SIR model (14) have locally mean square Hölder continuous martingale parts on $\mathbb {D}$ with Hölder exponent 0.5 with Hölder constant

$$ K_5=\frac{L_2^2}{L_1^2+L_2^2} ~\max _{|z|\le 1}\frac{e^{ 3 (L_1^2+L_2^2)z}-1}{z}. $$

### Proof

We have

$$\begin{aligned} {\begin{matrix} \displaystyle \mathbb {E}\!\Big [& \Big \Vert \!\int ^t_s \!\!\!\![g(X_{x_0,\!s}\!(u))\!-\!g(X_{y_0,\!s}\!(u))]dW\!(u)\Big \Vert ^2\Big ] = \!\int ^t_s \!\!\mathbb {E}\Big [\!\left\| g(X_{x_0,\!s}\!(u))\!-\!g(X_{y_0,\!s}\!(u))\right\| _F^2\!\Big ] du \\ & \le \; L_2^2 \!\int ^t_s \!\!\mathbb {E}\Big [\left\| X_{x_0,s}(u)-X_{y_0,s}(u)\right\| ^2\Big ] du \\ & \!\!\!{\mathop {\le }\limits ^{(26)}} \; 3~ L_2^2~ \Vert x_0-y_0\Vert ^2 \int ^t_s \!\!e^{ 3 (L_1^2+L_2^2)(u-s)}du = 3~ L_2^2~\frac{e^{ 3 (L_1^2+L_2^2)(t-s)}-1}{3 (L_1^2+L_2^2)}~\Vert x_0-y_0\Vert ^2 \\ & = \frac{L_2^2}{L_1^2+L_2^2} ~\frac{e^{ 3 (L_1^2+L_2^2)(t-s)}-1}{t-s}~(t-s)~\Vert x_0-y_0\Vert ^2. \end{matrix}} \end{aligned}$$

We may choose $\displaystyle r_{2}=0.5$ and define $\displaystyle K_5 := \frac{L_2^2}{\!L_1^2\!+\!L_2^2\!} ~\max _{|z|\le 1}\frac{\!e^{ 3 (L_1^2\!+\!L_2^2)z}\!-\!1\!}{z}< +\infty$. Hence

$$\begin{aligned} \displaystyle \mathbb {E}\Big [\left\| \int ^t_s\!\! \big [g(X_{x_0,s}(u))-g(X_{y_0,s}(u))\big ]~dW(u)\right\| ^2\Big ] \, \le \; K_5\Vert x_0-y_0\Vert ^2 (t-s). \end{aligned}$$

Therefore, the martingale part of *X* is locally Hölder-continuous in the mean square sense with the Hölder exponent $r_{sm}=0.5$ and Hölder constant

$$ K_5=\frac{L_2^2}{L_1^2+L_2^2} ~\max _{|z|\le 1}\frac{e^{ 3 (L_1^2+L_2^2)z}-1}{z}. $$

$$\square $$

Eventually, we can put our main results together and prove the convergence of approximations *Y* to exact solutions *X*. We shall measure how quickly that happens in terms of mean square convergence rates. That also means that our approximation (12) replicates the biologic relevant populations *S*, *I*, *R* well in the global sense.

### Theorem 10

(**Mean Square Convergence of ***Y*** to ***X*) Assume that the initial data $Y_0=X_0=(S_0,I_0,R_0) \in \mathbb {D}$ (a.s.) are independent of the $\sigma$-algebra generated by the underlying Wiener process *W*,

$$ \mathbb {E} \big [\Vert X_0\Vert _3^2\big ] < +\infty $$

and the hypothesis (31) is fulfilled with

$$\begin{aligned}&\sup _{(S,I,R) \in \mathbb {D}} \displaystyle \frac{|F_2(S,I,R)|}{R} + \sup _{(S,I,R) \in \mathbb {D}} |F_1(S,I,R)| \;<\; +\infty . \end{aligned}$$

Then, the numerical approximation *Y* governed by BIMs (12) with weights (13) is mean square convergent to the analytic solution *X* in $L^2(\Omega ,\mathcal {F},\{\mathcal {F}_t\}_{0 \le t \le T},\mathbb {P})$ with the global rate 0.5 for the stochastic SIR model (14) as the maximum mesh size $\Delta \downarrow 0$.

### Proof

The problem is well-posed in $L^2(\Omega ,\mathcal {F},\{\mathcal {F}_t\}_{0 \le t \le T},\mathbb {P})$ , i.e., it satisfies the conditions (A1)-(A8) on the domain

$$ \mathbb {D} = \Big \{(S,I,R)\in \mathbb {R}^3_+ : S>0,~I \ge 0, ~R>0,~ S+I+R \le K\Big \}. $$

justified by the details of Theorems 2-9. Hence, by Theorem 1, the numerical approximation *Y* governed by (12) with (13) converges to the analytic solution *X* (for all $Y_0=Y(0)=X_0 \in L^2$) as the maximum mesh size $\Delta \downarrow 0$.

Note that, the local moment *V*-boundedness is guaranteed with $V(x)=1\!+\!\Vert x\Vert ^2$. Indeed, we have

$$\begin{aligned} \mathbb {E}[V(X_0)]+\mathbb {E}[V(Y_0)]= &  \mathbb {E}[1+\Vert X_0\Vert ^2] +\mathbb {E}[1+\Vert Y_0\Vert ^2]\\= &  2+\mathbb {E}[\Vert X_0\Vert ^2]+\mathbb {E}[\Vert Y_0\Vert ^2] \;<\;\infty \end{aligned}$$

in view of all assumptions on the initial condition $X(0)=X_0=Y_0 \in \mathbb {D}$, and $\mathbb {E}[\Vert X_0\Vert ^2]<\infty$.

Moreover, $r_0=1.5$, $r_2=1$, and $r_{sm}=0.5$ satisfy the condition $r_0 \ge r_2 + r_{sm} \ge 1$. Consequently, for any fixed terminal time $T>0$, the global mean square convergence rate is $r_g=0.5$ by simple calculation $r_g \ge r_2+r_{sm}-1=1+0.5-1=0.5$ by Theorem 1. $\square$

## Numerical Analysis of Stochastic SIS Model

Similarly as before, we can mathematically justify our approximations for the stochastic SIS model (3). For this purpose, consider the balanced implicit method

39 $$\begin{aligned} Y_{n+1}=f(Y_n)\Delta _n +\sum _{j=1}^2 g^j(Y_n) \Delta W_n^j + c(Y_n) (Y_n-Y_{n+1}) \end{aligned}$$

with diagonal weights $\displaystyle c(Y_n)= A_n \cdot I_{2\times 2}$ with real $2\times 2$ unit matrix $I_{2\times 2}$ of $\mathbb {R}^{2 \times 2}$, where

40 $$\begin{aligned} A_n:=A(Y_n)=(\alpha \!+\!\gamma \!+\!\mu \!+\!\beta I_n)~\Delta _n\!+\! K~|F_1(Y_n)~\Delta W_n^1| \!+\! \frac{K}{S_n}~|F_2(Y_n)~\Delta W_n^2|. \end{aligned}$$

This relates to a discretization of the stochastic SIS model (in vector form)

$$\begin{aligned} \displaystyle dX(t)=f(X(t))~dt + g(X(t))~dW(t) \end{aligned}$$

where

$$\begin{aligned} \displaystyle X(t)= &  \begin{pmatrix} S(t)\\ I(t)\end{pmatrix}\text {,~~~} f(X(t))=\begin{pmatrix} -\beta S(t)I(t)+\mu (K-S(t))+\alpha I \\ \beta S(t)I(t)-(\alpha +\gamma +\mu )I(t)\end{pmatrix}, \\ g(X(t))= &  \begin{pmatrix} -S(t)I(t)F_1(X(t)) & I(t) F_2(X(t)) \\ S(t)I(t)F_1(X(t)) & -I(t) F_2(X(t))\end{pmatrix} \text {~~~and~~~} dW(t)=\begin{pmatrix} dW_1(t) \\ dW_2(t) \end{pmatrix}. \end{aligned}$$

Recall that the parameters $\alpha$, $\beta$, $\gamma$, $\mu$, and *K* are positive, and all diffusion rate functions $F_j:\mathbb {R}^2 \rightarrow \mathbb {R}$ are locally Lipschitz-continuous on the bounded domain (more precisely, a triangle in $\mathbb {R}^2$)

$$\begin{aligned} \mathbb {D}=\Big \{(S,I) \in \mathbb {R}^2\!: S>0,~I\ge 0,~ S+I\le K\Big \} \end{aligned}$$

with local Lipschitz-coefficients $\widetilde{L}_j$ on $\mathbb {D}$ ($j=1,2$). The $W_j$’s are standard Wiener processes defined on a complete probability basis $(\Omega ,\mathcal {F},\{\mathcal {F}_t\}_{t\ge 0},\mathbb {P})$ and are supposed to be independent of the initial value $X(0)=X_0=(S_0,I_0) \in \mathbb {R}^2$ with $\mathbb {E}[\Vert X_0\Vert ^2] < \infty$. The coefficients $f,g:\mathbb {R}^2 \rightarrow \mathbb {R}^2$ are locally Lipschitz-continuous on $\mathbb {D}$ and satisfy a linear growth condition on $\mathbb {D}$. That means that

$$\begin{aligned} \displaystyle \forall \, x,y \in \mathbb {D}\!: \quad \Vert f(x)-f(y)\Vert ^2\le &  L_1^2 \Vert x-y\Vert ^2,\\ \Vert g(x)-g(y)\Vert _{F}^2\le &  L_2^2\Vert x-y\Vert ^2,\\ \Vert f(x)\Vert ^2\le &  L_3^2 \Big (1+\Vert x\Vert ^2 \Big ),\\ \Vert g(x)\Vert _{F}^2\le &  L_4^2 \Big (1+\Vert x\Vert ^2 \Big ) \end{aligned}$$

$$\begin{aligned} \hbox {where}\quad L_1^2= &  \max \Big (10\beta ^2 K^2 +3 \mu ^2,10\beta ^2 K^2+3\alpha ^2 + 2(\alpha +\gamma +\mu )^2\Big ),\\ L_2^2= &  \sup _{(S,I)\in ~ \mathbb {D}} \Big ( 4 \widetilde{L}_1 K^4 + 8 F_1^2(S,I) K^2 + 4 \widetilde{L}_2 K^2 + 4 F_2^2(S,I) \Big ),\\ L_3^2= &  \max \Big ( 5 \beta ^2 K^2+ 3\alpha ^2+2(\alpha +\gamma +\mu )^2, 3\mu ^2 K^2, 3\mu ^2 \Big ),\\ L_4^2= &  \max \Big ( \sup _{(S,I) \in ~\mathbb {D}} 2 K^2 F_1^2(S,I), \sup _{(S,I)\in ~ \mathbb {D}} 2 F_2^2(S,I)\Big ) \end{aligned}$$

with local Lipschitz constants $\widetilde{L}_j$ of the diffusion rate functions $F_j$ on $\mathbb {D}$.

### Theorem 11

($\mathbb {D}$**-Invariance of ***Y*) Assume that the initial condition

$$Y_0=(S_0,I_0) \in \mathbb {D}=\Big \{(S,I)\in \mathbb {R}^2\!: S>0,~I \ge 0, ~ S+I \le K\Big \} \; (a.s.).$$

Then, the numerical solution *Y* governed by BIMs (39) with weights (40) is a.s. invariant with respect to $\mathbb {D}$ defined by (4) for all choices of step sizes $\{\Delta _n\}_{n \in \mathbb {N}}$.

### Proof

By rewriting the numerical method (39), we obtain

41 $$\begin{aligned} S_{n+1}= &  S_n + \Big [-\beta S_n I_n+\mu (K-S_n)+\alpha I_n\Big ]\Delta _n - S_n I_n F_1(Y_n) \Delta W_n^1 \nonumber \\ &  +I_n F_2(Y_n) \Delta W_n^2+ A_n(S_n-S_{n+1}), \end{aligned}$$

42 $$\begin{aligned} I_{n+1}= &  I_n + \Big [\beta S_n I_n - (\alpha +\gamma +\mu )I_n\Big ]\Delta _n + S_n I_n F_1(Y_n) \Delta W_n^1\nonumber \\ &  - I_n F_2(Y_n) \Delta W_n^2 +A_n(I_n-I_{n+1}). \end{aligned}$$

An equivalent explicit representation of (41) and (42) is

43 $$\begin{aligned} \!\!S_{n+1}\!\!=\! &  \! S_n \!\!+\!\frac{1}{1\!\!+\!\!A_n\!\!\!\!} \Big (\![-\beta S_n \!I_n\!+\!\mu (\!K\!\!-\!\!S_n\!)\!+\!\alpha I_n]\Delta _n \!-\!S_n \!I_n F_1\!(Y_n\!) \Delta \!W_n^1 \!+\!I_n F_2\!(Y_n\!) \Delta \!W_n^2\!\Big )\!,\quad \end{aligned}$$

44 $$\begin{aligned} \!\!I_{n+1}\!\!=\! &  \! I_n \!\!+\!\frac{1}{1\!\!+\!\!A_n\!\!\!} \Big (\![\beta S_n I_n \!-\! (\alpha \!+\!\gamma \!+\!\mu )I_n]\Delta _n \!+\!S_n I_n F_1(Y_n) \Delta \!W_n^1 \!\!-\! I_n F_2(Y_n) \Delta \!W_n^2 \!\Big )\!. \end{aligned}$$

We use induction on $n \in \mathbb {N}$. Assume that $S_0+I_0 \le K$, and $S_n+I_n \le K$ with $S_n>0$ and $I_n \ge 0$. Then, by adding up (44) and (44), we find that

$$\begin{aligned} \displaystyle S_{n+1}+I_{n+1}= &  S_n+I_n+ \frac{\mu \Delta _n}{1+A_n}\Big (K-S_n-I_n\Big )-\frac{\gamma I_n \Delta _n }{1+A_n}\\\le &  S_n+I_n+ \frac{\mu \Delta _n}{1+A_n}\Big (K-S_n-I_n\Big ) \\= &  \left( 1-\frac{\mu \Delta _n}{1+A_n}\right) (S_n+I_n)+ \frac{\mu \Delta _n}{1+A_n}K \\\le &  \left( 1-\frac{\mu \Delta _n}{1+A_n}\right) K+ \frac{\mu \Delta _n}{1+A_n}K \;=\; K~~\text {~~since~ } \frac{\mu \Delta _n}{1+A_n} \le 1. \end{aligned}$$

Positivity of the associated numerical solutions *Y* can be proven by substituting

$$A_n=(\alpha +\gamma +\mu +\beta I_n) \Delta _n\!+\! K |F_1(Y_n)\Delta W_n^1| \!+\! \frac{K}{S_n} |F_2(Y_n)\Delta W_n^2|$$

into (44). Therefore, one encounters

$$\begin{aligned} S_{n+1}= &  \frac{1}{1+A_n} \Big ( S_n+A_n S_n+[-\beta S_n I_n+\mu (K-S_n)+\alpha I_n]\Delta _n \\ &  -S_n I_n F_1(Y_n) \Delta W_n^1 +I_n F_2(Y_n) \Delta W_n^2\Big )\\= &  \frac{1}{1+A_n} \Big ( S_n+ (\alpha +\gamma +\mu +\beta I_n) S_n \Delta _n + K S_n |F_1(Y_n)\Delta W_n^1| \\ &  +\frac{K S_n}{S_n} |F_2(Y_n) \Delta W_n^2| + [-\beta S_n I_n+\mu (K-S_n)+\alpha I_n]\Delta _n \\ &  -S_n I_n F_1(Y_n) \Delta W_n^1 +I_n F_2(Y_n) \Delta W_n^2\Big ) \\= &  \frac{1}{1+A_n} \Big ( S_n+ (\alpha +\gamma ) S_n \Delta _n + K S_n |F_1(Y_n)\Delta W_n^1| \\ &  + K |F_2(Y_n) \Delta W_n^2| + \mu K S_n \Delta _n +\alpha I_n \Delta _n -S_n I_n F_1(Y_n) \Delta W_n^1\\ &  +I_n F_2(Y_n) \Delta W_n^2\Big ) ~>~ 0 \end{aligned}$$

and

$$\begin{aligned} I_{n+1}= &  \frac{I_n}{1+A_n} \Big (1+A_n+[\beta S_n - (\alpha +\gamma +\mu )]\Delta _n +S_n F_1(Y_n) \Delta W_n^1 - F_2(Y_n) \Delta W_n^2 \Big )\\= &  \frac{I_n}{1+A_n} \Big (1+(\alpha +\gamma +\mu +\beta I_n)~\Delta _n+ K |F_1(Y_n)~\Delta W_n^1| \\ &  +\frac{K}{S_n} |F_2(Y_n)~\Delta W_n^2|+[\beta S_n \!-\! (\alpha \!+\!\gamma \!+\!\mu )]\Delta _n \!+\! S_n F_1(Y_n) \Delta W_n^1 \!-\! F_2(Y_n) \Delta W_n^2 \Big )\\= &  \frac{I_n}{1+A_n} \Big (1+\beta (I_n+S_n)~\Delta _n+ K~|F_1(Y_n)\Delta W_n^1| + S_n F_1(Y_n) \Delta W_n^1 \\ &  - F_2(Y_n) \Delta W_n^2 + \frac{K}{S_n}|F_2(Y_n)\Delta W_n^2|\Big )\;\ge \;0 \text {~~since~ } \frac{K}{S_n} \;\ge \;1. \end{aligned}$$

Therefore, $\mathbb {P}(\{Y_n=(S_n,I_n) \in \mathbb {D}\})=1$ for all $n \ge 1$. $\square$

Finally, we may justify the global convergence of our approximations (39) of SIS model (3).

### Theorem 12

(**Mean Square Convergence of ***Y*** to ***X*) Assume that the initial data $Y_0=X_0=(S_0,I_0) \in \mathbb {D}$ (a.s.) are independent of the $\sigma$-algebra generated by the underlying Wiener process $W\!$, $\mathbb {E} \big [\Vert X_0\Vert _2^2\big ] < +\infty$ and

45 $$\begin{aligned} \sup _{(S,I) \in \mathbb {D}} \displaystyle \frac{|F_2(S,I)|}{S} + \sup _{(S,I) \in \mathbb {D}} |F_1(S,I)| \;<\; +\infty . \end{aligned}$$

Then, the numerical approximation *Y* governed by BIMs (39) with weights (40) is mean square convergent to the analytic solution *X* in $L^2(\Omega ,\mathcal {F},\{\mathcal {F}_t\}_{0 \le t \le T},\mathbb {P})$ with the global rate $r_g=0.5$ for the stochastic SIS model (3).

### Proof

The problem is well-posed in $L^2(\Omega ,\mathcal {F},\{\mathcal {F}_t\}_{0 \le t \le T},\mathbb {P})$, i.e., it satisfies assumptions (A1) - (A8) on the domain

$$\begin{aligned} \mathbb {D}=\Big \{(S,I)\in \mathbb {R}^2_+\!: S>0,~I\ge 0,~ S+I\le K\Big \}. \end{aligned}$$

Because the numerical approximation is a.s. invariant with respect to $\mathbb {D}$ under (45) by Theorem 11, the coefficients *f* and *g* are locally Lipschitz-continuous and satisfy a linear growth condition on the same domain $\mathbb {D}$. Mean and mean square consistency of *Y* to *X* can be proven due to the invariance of *Y* on $\mathbb {D}$ and condition (45) (i.e. by similar proofs as for Theorems 7 and 8 for the SIR model). Consequently, all assumptions of Theorem 1 are fulfilled in order to conclude the global rate of convergence $r_g \ge 0.5$. The proof-steps are very similar to that of the stochastic SIR model. Thus, we refrain from listing all the details here. $\square$

## Report on Simulations for SIS and SIR Models

### Simulation Results for a SIS Model

In order to graphically illustrate some of our results, we simulate the solution of stochastic SIS model (3) by using the discretization *Y* based on the balanced implicit method (39) with weights (40). Under some appropriate assumptions, stochastic asymptotic stability of the disease free and the endemic equilibria are proved in Schurz (2019). First, we consider the model with time measured in portion of a full calendar year (365 days) and

(i) $\mu =$ birth rate $=$ natural death rate $= 1/75 = 0.013$ corresponding to a human life expectancy of 75 years,
(ii) $\beta = 0.02$ explaining that average infectives makes contact sufficiently to transmit infection with 0.02*K* others per year, where $K=200$,
(iii) $\alpha = 13$ corresponding to infectives recover after a mean infective period of 1/13 year (4 weeks),
(iv) $\gamma = 13$ describing a disease from which infectives die because of the disease after a mean period of 1/13 year.

Therefore, the related stochastic SIS model is given by

46 $$\begin{aligned} dS= &  \Big (\!-0.02 S I +0.013(200-S)+13 I \!\Big ) dt - S I F_1(S,I)~dW_1+ I F_2(S,I)~dW_2,\nonumber \\ dI= &  \Big (\!0.02 S I-(13+13+0.013)I\!\Big ) dt + S I F_1(S,I)~dW_1 - I F_2(S,I)~dW_2 \end{aligned}$$

where the diffusion rate functions $F_j$’s are specified below. This SIS model (46) is discretized by BIMs (39) with weights (40) for all simulation results below.

**Fig. 1:**
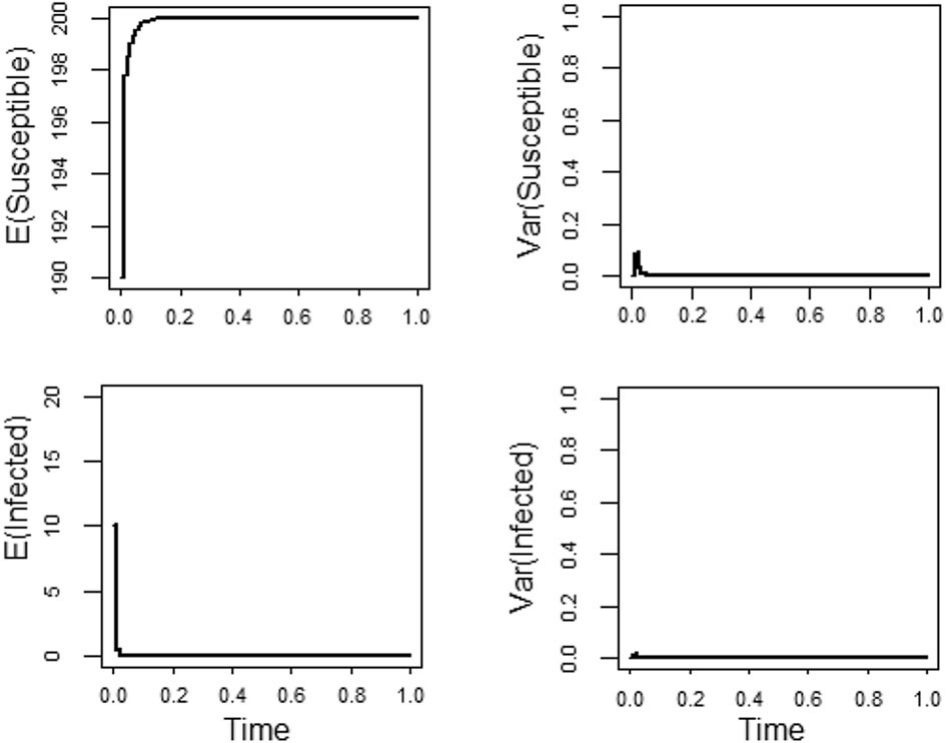
Simulations of the stochastic SIS model with the diffusion rate functions $F_1(S,I)=\frac{S-S_2}{K^2}$ and $F_2(S,I)= S \frac{I-I_2}{K}$. Here we have used the initial value $(S_0,I_0)=(190,10)$ and the uniform step size $\Delta =10^{-2}$ in simulations with the parameters $\alpha =13$, $\beta =0.02$, $\gamma =13$, $\mu =0.013$, and $K=200$. Expectations and variances are estimated over samples of 10000 trajectories.

In Schurz (2019), we proved that the disease free equilibrium $(S,I)=(K,0)$ of (3) is stochastically asymptotically stable if the basic reproduction number $\displaystyle \mathcal {R}_0=\frac{\beta K}{\alpha +\gamma +\mu }<1.$ The left two pictures of Figure 1 show the dynamics of expected values of Susceptible and Infected for the diffusion rate functions $F_1(S,I)=\frac{S-S_2}{K^2}$ and $F_2(S,I)=S \frac{I-I_2}{K}$, where $(S_2,I_2)=\left( \frac{K}{\mathcal {R}_0},\frac{\mu K}{\gamma +\mu }\left( 1-\frac{1}{\mathcal {R}_0}\right) \right)$. As expected (since $\mathcal {R}_0=0.15<1$), the trajectories of the solution *Y* settle around the disease free equilibrium (200, 0). The last two pictures display the evaluation of the variances of Susceptible and Infected. As it is seen, fairly small variances occur and they rapidly go to zero. Hence, the non-random disease free equilibrium is approached as the integration time *t* advances.

**Fig. 2:**
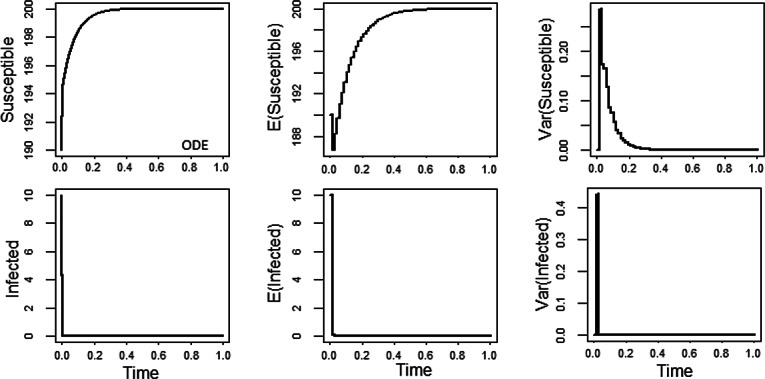
Numerical solutions of stochastic SIS model by BIMs (39) for diffusion rate functions $F_1(S,I)=\sin (S+I)$ and $F_2(S,I)= \frac{I^2}{K} I_{S>0.01}$, where $\alpha =13$, $\beta =0.02$, $\gamma =13$, $\mu =0.013$, $K=200$, and the initial values $(S_0,I_0)=(190,10)$ while using the uniform step size $\Delta =10^{-2}$. In simulations, the expectations are estimated over samples of 10000 trajectories.

Figure 2 (4 plots at the right side) illustrates the stochastic asymptotic stability of the disease free equilibrium (*K*, 0) for a set of diffusion rate functions $F_1$ and $F_2$, which is different from that of Figure 1. For comparison, we also plot the solutions *S*(*t*) and *I*(*t*) of the associated non-random ODE with absent noise terms versus time (see left two plots in Figure 2). The middle pair of plots portrays the dynamics of the expected values of Susceptible and Infected populations reaching the states of the disease free equilibrium quickly, whereas the far right hand pair displays fast vanishing variances versus time after some initial fluctuations (needed to adjust to the stationary regime). For those simulations, we have considered the same parameter set $\alpha =13$, $\beta =0.02$, $\gamma =13$, $\mu =0.013$, and $K=200$ for the model (46) as in Figure 1, but we make use of the set of $F_j$’s defined by $F_1(S,I)=\sin (S+I)$ and $F_2(S,I)= \frac{I^2}{K} I_{S>I}$. Clearly, those functions $F_k$ obey the assumptions (45) of the mean square convergence theorem 12 of our BIMs (39) with weights (40). Note that the functions $F_j$ are local Lipschitz continuous with exceptional set of 2D Lebesgue measure zero. Figure 2 shows that the simulations agree with the analytic results of the SI model of Schurz (2019) on the stochastic stability of the disease free equilibrium (*K*, 0) since trajectories of solutions of stochastic system (46) approach the disease free equilibrium (*K*, 0) as time *t* advances.

Under certain conditions (on the basic reproduction number and the diffusion rate functions $F_1$ and $F_2$), the endemic equilibrium $(S_2,I_2)\!=\!\left( \!\frac{K}{\mathcal {R}_0},\!\frac{\mu K}{\gamma +\mu }\!\left( 1-\frac{1}{\mathcal {R}_0}\!\right) \!\right)$ of (3) is stochastically asymptotically stable (cf. Schurz (2019)). The major conditions are as follows.

1. The basic reproduction number $\displaystyle \mathcal {R}_0=\frac{\beta K}{\alpha +\gamma +\mu }$ satisfies $\displaystyle \mathcal {R}_0>1$ and
2. $$-\mu (S\!-\!S_2)^2 -(\gamma \!+\! \mu ) (I\!-\!I_2)^2 +\frac{ (2\mu \!+\!\gamma ) I_2}{2 \beta } \Big (\!S^2 F_1^2(S,I)\!+\! F_2^2(S,I)\!\Big ) ~ \mathbf {<} ~0 $$ for $$F_j(S_2,I_2)\!=\!0.$$

This time we consider the following set of parameters: $\alpha =52$ corresponding to infectives recover after a mean infective period of one week, contact rate $\beta =0.03$, disease related death rate $\gamma =13$, birth date and natural death rate $\mu =0.013$, and the carrying capacity of the population $K=3000$ in (3). Hence, the basic reproduction number $\displaystyle \mathcal {R}_0=1.38>1$. Moreover, the diffusion rate functions $F_1(S,I)=\frac{1}{K^2}(S-S_2)$ and $F_2(S,I)=\frac{S}{K}(I-I_2)$ are considered.

Note that the conditions for asymptotic stability of the endemic equilibrium $(S_2,I_2)=\left( \frac{K}{\mathcal {R}_0},\frac{\mu K}{\gamma +\mu }\left( 1-\frac{1}{\mathcal {R}_0}\right) \right) =\left( 2167.1, 0.8321\right)$ are satisfied for our parameters.

**Fig. 3:**
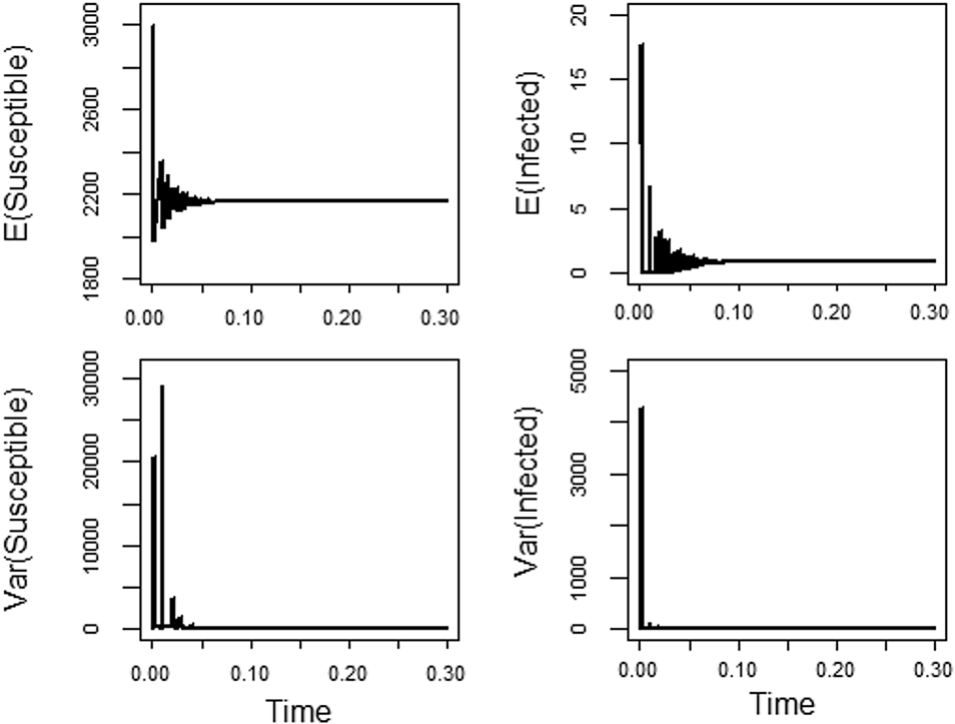
For the parameters $\alpha \!=\!52$, $\beta \!=\!0.03$, $\gamma \!=\!13$, $\mu \!=\!0.013$ and $K\!=\!3000$, the endemic equilibrium $(S_2,I_2)\!=\!(2167.1, 0.8321)$ is stochastically asymptotically stable. In those simulations, the initial value $(S_0,I_0)=(2990,10)$, uniform step size $\Delta =10^{-3}$ and sample size 10000 are used.

In the top two pictures of Figure 3, the expected values of Susceptible and Infected populations versus time are plotted. They show that Susceptible and Infected populations quickly settle around the equilibria (in average). The bottom two pictures display the evaluation of the variances of Susceptible and Infected populations versus time.

**Fig. 4:**
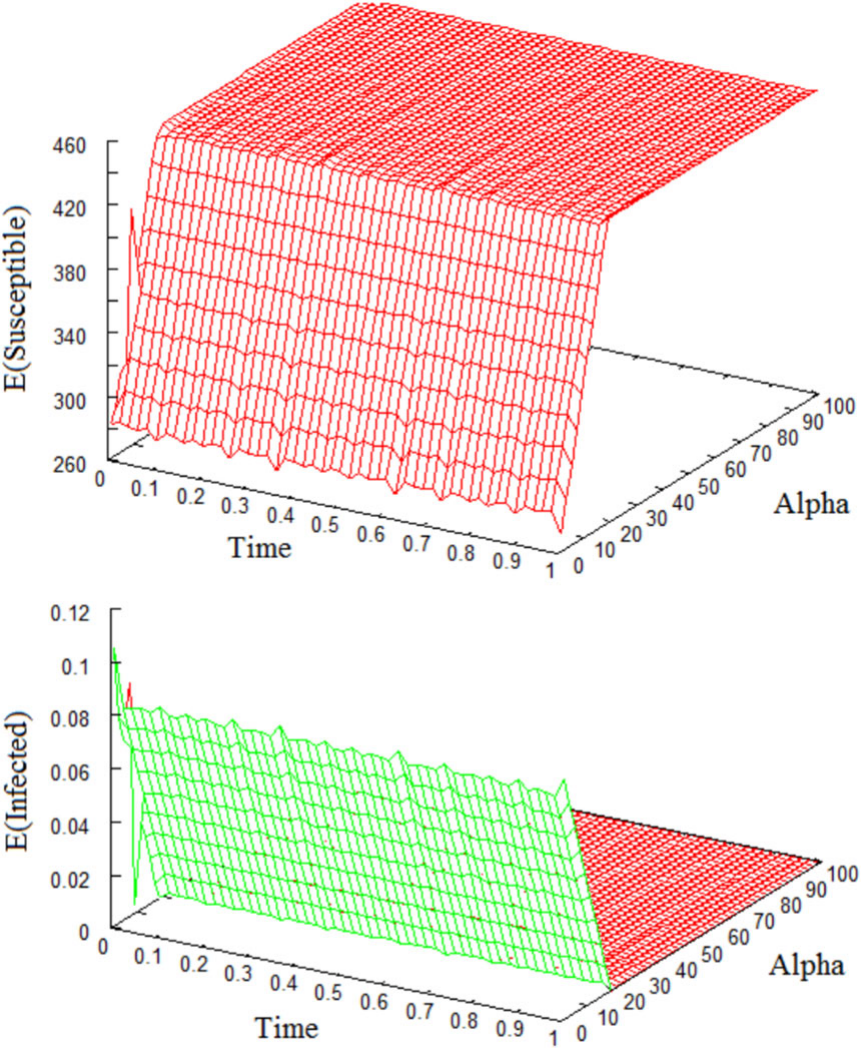
Expectations of Susceptibles and Infectives versus $\alpha$, time *t*, using fixed $\beta \!=\!0.1$, $\gamma \!=\!26$, $\mu \!=\!0.013$, $K\!=\!450$. If $\alpha \!\ge \! 19$ then $\mathcal {R}_0 \!=\!\frac{45}{26.013+\alpha } \!<\! 1$, and there exists only one equilibrium $(S,I)\!=\!(450,\!0)$, which is stochastically asymptotically stable. If $\alpha \!\le \! 18$ then $\mathcal {R}_0 \!>\! 1$. Hence, an endemic equilibrium $\displaystyle (S,I)\!=\! \left( 260.13\!+\!10 \alpha , 0.095\!-\!0.005 \alpha \right)$ is stochastically asymptotically stable. The initial value $(S_0,I_0)\!=\!(440,\!10)$, uniform step size $\Delta \!=\!10^{-3}$ and sample size of 10000 are used.

In the next two figures, we use the diffusion rate functions $F_1(S,I)=\frac{1}{K^2}(S-S_2)$, $F_2(S,I)=\frac{S}{K}(I-I_2)$ and the carrying capacity $K=450$ in (3). In Figure 4, we fix all parameters $\beta =0.1$, $\gamma =26$, $\mu =0.013$ (except for the recovery rate $\alpha$) and plot the expected values of Susceptible and Infected versus time versus the recovery rate $\alpha$. These pictures show the effects of the recovery rate on the asymptotic stability of equilibria. If $\alpha$ gets large then we lose the existence of an endemic equilibrium and verify stochastic asymptotic stability of the disease free equilibrium of the system (46).

**Fig. 5:**
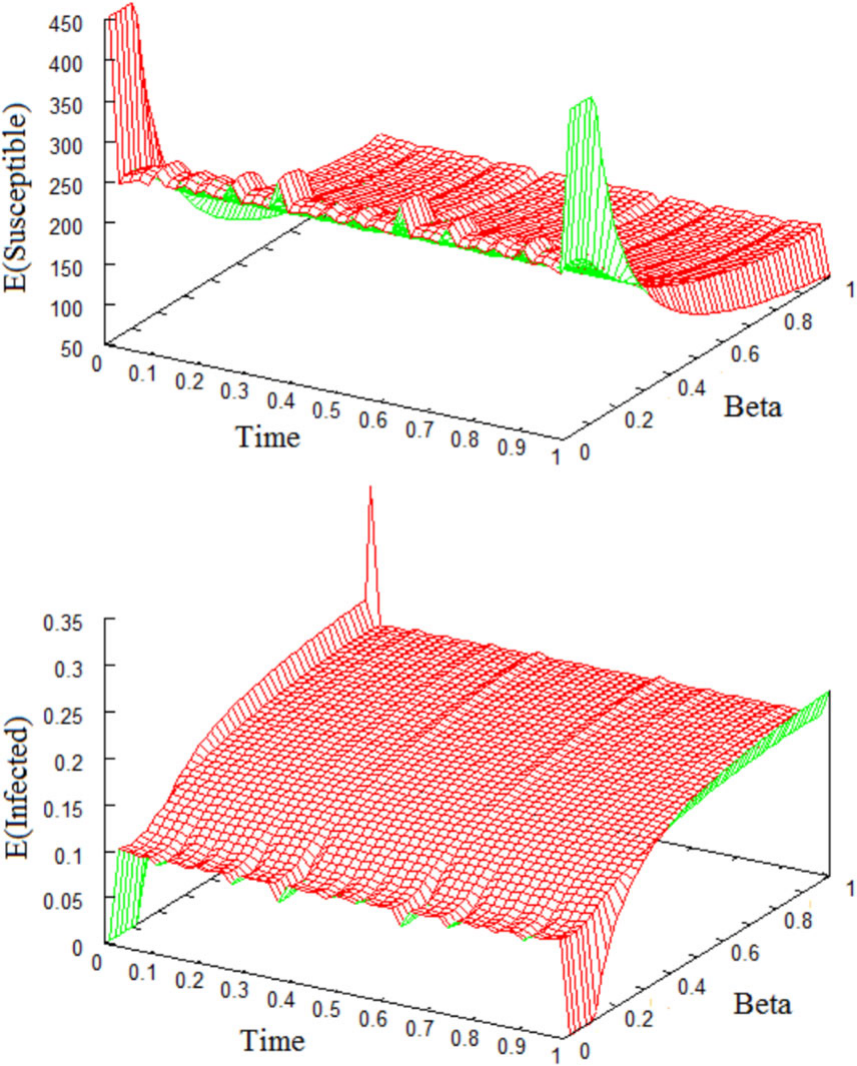
Expectations of Susceptibles and Infectives versus $\beta$, time *t* with fixed $\alpha \!=\!26$, $\gamma \!=\!26$, $\mu \!=\!0.013$, $K\!=\!450$. If $\beta \!\le \! 0.115$ then $\mathcal {R}_0 \!=\!8.652 \beta \!<\!1$, and there exists only one equilibrium $(S,I)\!=\!(450,\!0)$ which is stochastically asymptotically stable. If $\beta \!\ge \! 0.116$ then $\mathcal {R}_0 \!>\! 1$. Hence, the endemic equilibrium $\displaystyle (S,I)\!=\! \left( \!\frac{52.013}{\beta }, 0.225-\frac{0.0260}{\beta } \!\right)$ is stochastically asymptotically stable. The trajectories started with $(S_0,I_0)\!=\!(440,\!10)$, using uniform step size $\Delta \!=\!10^{-3}$ and samples over 10000 trajectories.

Similarly in Figure 5, we fix all parameters ($\alpha =26$, $\gamma =26$, $\mu =0.013$) other than the contact rate $\beta$ and plot the expected values of Susceptible and Infected populations versus time *t* versus $\beta$. These pictures show the effect of $\beta$ on the asymptotic stability of equilibria. If $\beta$ gets small then we lose the existence of an endemic equilibrium and verify the stochastic asymptotic stability of the disease free equilibrium.

**Fig. 6:**
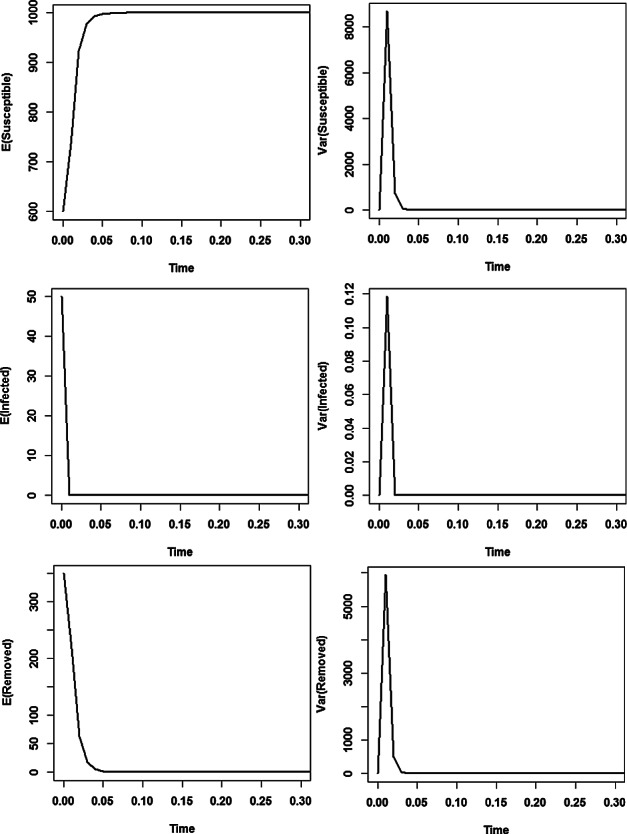
Expectations and Variances of Susceptibles and Infectives versus time *t* with Asymptotically Stable Disease Free Equilibrium (*K*, 0, 0) and $F_2(S,I,R)=I \!\cdot \! I_{\{R>0.01\}} / K^2$, $F_1(S,I,R)=(S-K)/K^3$ with K=1000, $\alpha =52$, $\beta = 0.05$, $\gamma =52$, $\mu =0.013$ and $\beta K/\gamma =0.96<1$. The paths started at $(S_0,I_0,R_0)\!=\!(600,\!50,\!350)$ and were generated by BIMs (12) using uniform step size $\Delta \!=\!10^{-2}$. Statistical averaging is conducted over 10000 sample paths.

### A Simulation of a Stochastic SIR Model

Eventually, we provide some simulation results for the SIR model (1). Consider

47 $$\begin{aligned} dS= &  \Big (\!-\beta S I +\mu (K-S)\!\Big ) dt - \frac{1}{K^3} S I \!\cdot \!(S-S^*) dW_1\nonumber \\ dI= &  \Big (\!\beta S I\!-\!\big (\!\alpha \!+\!\gamma \!+\!\mu \!\big )\!I\!\Big ) dt \!+ \!\frac{1}{K^3} S I \!\cdot \! (S\!-\!S^*) dW_1 \!-\!\frac{I}{K^2}\!\cdot \! I_{\{R>0.01\}}\!\cdot \! (I\!-\!I^*) dW_2 \nonumber \\ dR= &  \Big (\!\alpha I - \mu R\!\Big ) dt+ \frac{I}{K^2}\!\cdot \! I_{\{R>0.01\}}\!\cdot \! (I-I^*) dW_2 \end{aligned}$$

with equilibrium $\displaystyle \!(\!S^*\!\!,\!I^*\!\!,\!R^*\!)\!\!=\!\! \left( \!\!\frac{K}{\mathcal {R}_0\!\!}, \!\frac{\mu }{\beta \!}\!\left( \mathcal {R}_0\!-\!\!1\!\right) \!,\! \frac{\alpha }{\beta \!}\!\left( \mathcal {R}_0\!-\!\!1\!\right) \!\!\right) \!$, reproduction number $\displaystyle \!\mathcal {R}_0\!\!=\!\!\frac{\beta K}{\!\!\alpha \!+\!\gamma \!+\!\mu \!\!\!},$

(i) $K=1000$ is a carrying capacity, i.e., the maximum population size.
(ii) $\mu$ is a per capita death rate (birth rate) per unit time. We take $\mu =1/75=0.013$ corresponding to a human life expectancy of 75 years. Hence, $\mu K$ is the number births in a year (13 births for this example) and $\mu S$ the number of natural deaths in the susceptible population.
(iii) $1/{\alpha }$ is the mean of the infective period. We used $\alpha =52$ and 13 corresponding to infectives recover after a mean infective period of 1/52 and 1/13 year (1 and 4 weeks), resp.
(iv) $1/{\gamma }$ is the mean of the disease related death period. Below, we use $\gamma = 52$ and 26 describing a disease from which infectives die because of the disease after a mean period of 1/52 and 1/26 year (1 and 2 weeks), resp.
(v) $\beta$ is an infection rate (contact rate), $~\beta S I$ is the number of new infectives in a year. Below, we use $\beta =0.05$ and 0.1 such that average infectives make sufficient contacts to transmit the infection with $0.05 K=50$ and $0.1 K=100$ in a calendar year.

Now, we simulate this SIR model (47) with contact diffusion rates

$$ F_1(S,I,R) = \dfrac{S-S^*}{K^3} \quad \hbox {and} \quad F_2(S,I,R) = \dfrac{I-I^*}{K^2} I_{\{R>0.01\}} $$

where $I_{\{\bullet \}}$ denotes the indicator function that $R>0.01$. They fulfill the assumptions of our previous Theorems on consistency and convergence of balanced implicit methods (12) on the bounded domain $\mathbb {D}\subset \mathbb {R}_+^3$ of the form (2). Note that the subset of discontinuities of $F_2$ in $\mathbb {D}$ (where $F_2$ is not locally Lipschitz continuous) has 2D Lebesgue measure zero. Hence, the essential supremum of $F_2$ over $(S,I,R) \in \mathbb {D}$ is finite. Therefore, we are entitled to apply our numerical methods to the SIR model (47) whenever $(S_0,I_0,R_0) \in \mathbb {D}$.

Figure 6 displays the mean and variances of the Susceptibles *S*(*t*), Infected *I*(*t*) and Recovering populations *R*(*t*) versus time. We clearly reckon that the solutions (*S*(*t*), *I*(*t*), *R*(*t*)) reach the disease free equilibrium $(K,0,0)=(1000,0,0)$ as the reproduction number $\mathcal {R}_0<1$ for our data.

**Fig. 7:**
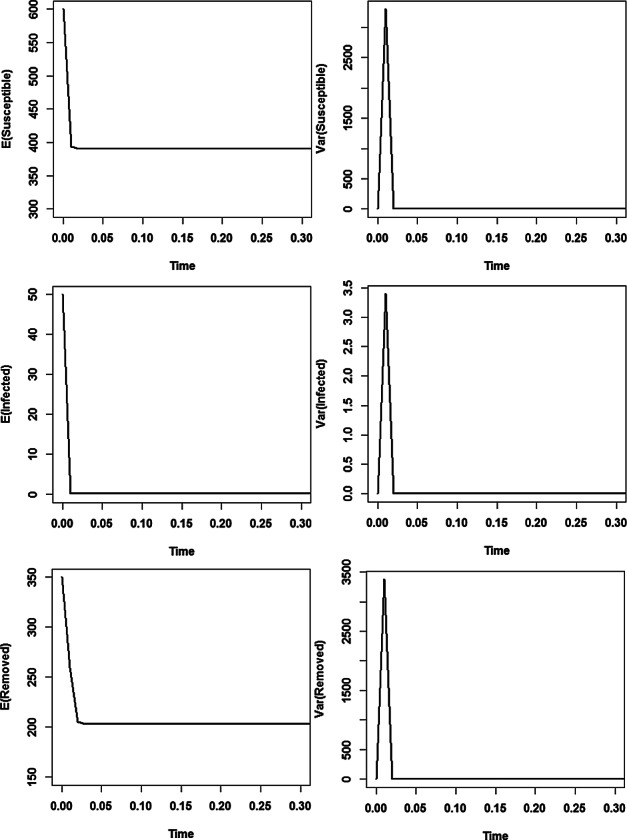
Expectations and Variances of Susceptibles and Infectives versus time *t* with Asymptotically Endemic Equilibrium $(S^*,I^*,R^*) \approx (390.13,0.20,203.32)$ and $F_2(S,I,R)= (I-I^*)_{\{R>0.01\}}/K^2$, $F_1(S,I,R)=(S-S^*)/K^3$ with K=1000, $\alpha =13$, $\beta = 0.1$, $\gamma =26$, $\mu =0.013$ and reproduction number $\mathcal {R}_0=2.56>1$. All paths started at $(S_0,I_0,R_0)\!=\!(600,\!50,\!350)$ and were generated by BIMs (12) using uniform step size $\Delta \!=\!10^{-2}$. Statistical averaging is conducted over 10000 sample paths.

Figure 7 portrays the mean and variances of the Susceptibles *S*(*t*), Infected *I*(*t*) and Recovering populations *R*(*t*) versus time. We clearly reckon that the solutions (*S*(*t*), *I*(*t*), *R*(*t*)) reach the endemic equilibrium $(S^*,I^*,R^*) \approx (390.13,0.20,203.32)$ as time *t* advances as the reproduction number $\mathcal {R}_0>1$ for our data.

Figures 6 and 7 confirm the stability results of Schurz and Tosun (2015) by our positive, invariant, stable, consistent and convergent numerical methods (12) for the SIR model (47), which are asymptotically reaching the correct, predicted equilibria, resp. Consequently, the simulation results of this section provide a "statistical evidence of the dynamic consistency" of our numerical methods.

## Conclusion

This paper presents a mathematical justification (i.e., executing the concept of Dynamic Consistency) of simulations carried out by Balanced Implicit Methods (BIMs) with appropriate weights (i.e. diagonal matrices) for stochastic SIR and SIS models with variable, local Lipschitz-continuous diffusion contact rates $F_j$ on bounded domains $\mathbb {D}$ as provided in Schurz (2019), and Schurz and Tosun (2015). Dynamic consistency means that qualitative properties of both exact and numerical solutions coincide. In Schurz and Tosun (2015), the unique existence of positive and uniformly bounded solutions of SIR models (1) with variable diffusion contact rates $F_j$ on bounded domains $\mathbb {D}\subset \mathbb {R}_+^3$ of the form (2) and criteria for asymptotic stability of disease free and endemic equilibria have been established. The asymptotic behavior of positive analytic solutions to another stochastic SIR model is also investigated in Jianga et al. (2011), but not our SIR model with general diffusion rate functions. Guo et al. (2018) have tried to construct positive numerical solutions for a special class of SIRS models with specific diffusion rates $\sigma S I/(1+\alpha I^2)$ (based on Euler-Maruyama method), but not for more general rate functions $F_j$. Their methods failed to preserve positivity for any step size $\Delta$. Ruan and Wang (2003) have used a specific class of our BIMs Milstein et al. (1998) to preserve positivity and convergence to the analytic solutions of specific SIRS models, but they did not establish results for models with general diffusion rate functions and neither on uniform boundedness. Feng and Wang (2020) prove the positivity of our BIMs (cf. Schurz (1997)) for SIRS models with specific diffusion rates $\sigma S I/(1+\alpha I^2)$ in *S* and *I* populations, but no noise in *R* population. They did not prove uniform boundedness on bounded prisms in $\mathbb {R}^3$. In Schurz (2019), the unique existence of positive and uniformly bounded solutions of SIS models (3) with variable diffusion contact rates $F_j$ on bounded domains $\mathbb {D}\subset \mathbb {R}_+^2$ of the form (4) and criteria for asymptotic stability of disease free and endemic equilibria are verified. Our numerical solutions based on appropriate BIMs possess those properties justified by the convergence result of Theorem 10 for the SIR model (1) and Theorem 12 for the SIS model (3). Moreover, the uniform boundedness of BIMs is established by Theorem 2 for SIR model (1) and Theorem 11 for SIS model (3). Consequently, the dynamic consistency of BIMs for those models is confirmed. Besides, local consistency rates for SIR models (1) are obtained by Theorems 7 and 8. Same consistency rates are valid for SIS models (3).

The numerical challenge is that, despite the presence of unbounded random noise in those models, to generate simulations which exclusively live on bounded domains $\mathbb {D}\subset \mathbb {R}^3_+$ (such as positive prisms or compact sets) for all time-step sizes $\Delta _n$ for their biological relevance. Other standard models only provide us (a.s.) positivity of populations for *S*, *I*, *R*, but which are not uniformly bounded from above or using very complex-restricted time-step sizes $\Delta _n$ or $\Delta _n$ which depend on actual randomness in an anticipating manner (We indeed do not need such restrictions). However, the biological reality forces us to model rather on bounded, nonrandom domains $\mathbb {D}$ for *S*, *I*, *R* as the total population size $N(t)=S(t)+I(t)+R(t)$ is always uniformly bounded by some nonrandom positive constant due to limited, finite natural resources. We provide fairly easily implementable numerical methods with a specific class of Balanced Implicit Methods (12) and (39) for those models, resp. The primary task here was not to validate the model in terms of biologically relevant data, but rather to justify the dynamic consistency (consistency, stability, convergence, positivity, (a.s.) uniform boundedness on $\mathbb {D}$) of appropriate numerical methods (12) and (39) for "less expensive" simulation studies of those epidemic models with noisy variable contact rates within a biologically relevant context. The alternative would be to use numerical methods with truncated noise. However, the truncation need to be non-anticipating because the Itô calculus which is relevant for biological model interpretations. Moreover, the random numerical truncation techniques are not quite clearly represented in multi-dimensional domains $\mathbb {D}$ relevant to our stochastic SIR and SIS models by the literature. Our methods guarantee a.s. boundedness on and invariance of $\mathbb {D}$, consistency and convergence to the exact solutions on any finite time-interval without any needed (possibly random) truncation procedure - a fact, which is important for correct path-wise considerations and which can be continued for very large time-intervals without further restrictions (e.g., on associated moments of random variables or variable time-step sizes $\Delta _n$). Despite non-globally Lipschitz-continuous coefficients of our SDE models, we are able to prove the global mean square convergence rate $r_g \ge 0.5$ of our methods on bounded prisms. To the best of our knowledge, a justification of numerical methods for stochastic SIR (1) and SIS models (3) with general variable diffusion rate functions $F_j$ on uniformly bounded domains $\mathbb {D}\subset \mathbb {R}^3_+$ has not been done in that depth (apart from few, very special cases of $F_j$’s). The key to our analysis is that we constructed and verified $\mathbb {D}$-invariant, mean and mean square consistent numerical methods for stochastic SIR and SIS models, whereas the most common (standard) numerical methods such as Euler, Milstein, and Taylor methods fail to possess either positivity or uniform boundedness. For example, Schurz (1996) has shown that Euler-Maruyama method cannot preserve (a.s.) positivity for simplest test equations with multiplicative noise whatever uniform step size $\Delta$ one uses. Of course, standard methods with locally Lipschitz-continuous coefficients produce locally consistent approximations. However, their invariance w.r.t. bounded domains $\mathbb {D}$ has not been shown for non-random or truly adapted step sizes $\Delta _n$. Therefore, rigorous proofs of global convergence of them are missing (without additional truncation procedures, with the side-effect destroying their global convergence or changing their path-wise long-term behavior decisively). The key is to understand and improve their stability and invariance behavior in the future. In passing, it is worth noting that the discussion and first results on the positivity and uniform boundedness of stochastic numerical methods for SDEs has began with the contribution of Schurz (1996) in the literature. Since then, many researcher have picked up those original ideas and modified them. Positivity, boundedness and invariance of our simulation methods on the correct, bounded domains $\mathbb {D}$ is important for the qualitative replication of the dynamics of stochastic SIR and SIS models and drawing adequate, biologically relevant conclusions on them (for original ideas in $\mathbb {R}^1_+$, see Schurz (1996, 1997, 2007)). Note that those domains $\mathbb {D}$ geometrically relate to prisms in $\mathbb {R}^3_+$ or $\mathbb {R}^2_+$, resp. Remarkably, our simulation methods based on BIMs provide invariant numerical approximations for those prisms without any local projections onto $\mathbb {D}$ or local truncation of unbounded noise increments, whereas both alternatives are causing additional numerical errors. The numerical invariance of prisms $\mathbb {D}$ is guaranteed by our $L^2$-convergent simulation methods by choosing appropriate weights (13) and (40), resp. However, the choice of weights $A_n$ for the BIMs must be taken carefully. For the sake of simplicity, we have chosen diagonal matrices here. This paper represents an extension of 1D stochastic logistic growth models (presented and analyzed in Schurz (2007)) and their adequate discretization on $\mathbb {D}\subset \mathbb {R}^1$ to prisms in $\mathbb {R}^3$ (for SIR model) and $\mathbb {R}^2$ (for SIS model).

Simulation results for the stochastic SIS model are found in this paper or also in Schurz (2019), and, for the stochastic SIR model, also in Schurz and Tosun (2015). For stochastic SEIR models, one can also consult simulation results in Chandrasena and Chandrasena (2024) and Chandrasena et al. (2025), based on appropriate BIMs.

Due to the complexity, we have not studied oscillation theory of related stochastic systems by this paper. In fact, we have noticed some quasi-oscillations in the plots of Figure 3. This relates to the open question whether one sees here spurious quasi-oscillations of the used numerical method *Y* or indeed quasi-oscillations of the underlying exact solution $X=(S,I)$ or $X=(S,I,R)$. However, the theory of stochastic numerical quasi-oscillations is very undeveloped. So, this is a good open question of qualitative relevance of stochastic numerical methods to be investigated in the future. Anyway, in the limit maximum step sizes $h_{max}$ of balanced implicit methods tend to zero, the plots should coincide with that of exact solutions *X* by our convergence results. Unfortunately, we can not do this in the real world (due to the "computational and statistical finiteness" of our world).

## Data Availability

Data sharing is not applicable to this article as no real sample data sets were analyzed during the studies. It is an entirely theoretical research article. However, electronic data of the simulations are available from the authors upon reasonable request.

## Appendix A Most Used Parameters and Variables

For the convenience of the reader, we state a table of most used variables and parameters here.Table 1 This supplement relates to the presented and studied stochastic SIR and SIS models.

**Table 1:** Model Variables and Their Biologic Meaning.

Variable	Biological Meaning
$\mathcal {R}_0$	basic reproduction number
*S*	portion of susceptible population
*I*	portion of infected population
*R*	portion of removed population
*N*	total population number
$\beta$	contact rate
$\mu$	natural death rate
$\alpha$	recovery rate
$\gamma$	disease related death rate
$F_j$	diffusion rate functions (environmental noise intensities).

## Appendix B A Note on Biological Interpretations

The main properties, which we investigated to justify the adequateness of our numerical methods (BIMs) for simulation of stochastic SIR and SIS models, have the following biologic interpretations: The positivity of BIMs implies exclusive positive portions of susceptible $S_n$, infected $I_n$ and removed individuals $R_n$ at each time $t_n$. The invariance of BIMs w.r.t. bounded domains $\mathbb {D}$ guarantees that the vector $(S_n,I_n,R_n)$ exclusively stays in $\mathbb {D}$ as time $t_n$ progresses. That is supported by the fact that limited resources for each individuals and competition between them force the total population to stay in a bounded domain, independent of fluctuating components of their environment. Stability of BIMs ensures that the population $(S_n,I_n,R_n)$ stays close to the equilibria as time $t_n$ advances and cannot exponentially diverge from them. Consistency of BIMs yields that the numerical dynamics locally approximate the underlying continuous time solutions and controls the accuracy by the size of time-step sizes. Hölder continuity measures how smooth the solution paths (*S*, *I*, *R*) can be expected to be. Convergence of BIMs asserts that the approximate solutions $(S_n,I_n,R_n)$ converge to the exact solutions (*S*, *I*, *R*)(*t*) as long as $t_n$ tends to *t* and associated maximum of step sizes $t_k-t_{k-1}$ vanish to zero. Finally, contractivity of BIMs implies that solutions $(S_n,I_n,R_n)$ for neighbored initial values $(S_0,I_0,R_0)$ must stay close each other and cannot deviate too much as time $t_n$ evolves. So, those concepts are very reasonable and support the adequateness simulations to related biologically relevant patterns.

## Appendix C Auxiliary Lemma For Proof of THM 6

### Lemma 1

Assume that $x_0=(s_0,i_0,r_0)\in \mathbb {D}$ and

$$\begin{aligned} A(t,\!s)&= (\alpha \!+\!\gamma \!+\!\mu \!+\!\beta i_0) |s\!-\!t| \!+\! K |F_1\!(x_0)[W_1\!(t)\!-\!W_1\!(s)]|\\ &\quad + \frac{K}{r_0} |F_2\!(x_0)[W_2\!(t)\!-\!W_2\!(s)]|. \end{aligned}$$

Then, for all nonrandom *t*, *s*, we have

$$\begin{aligned}&\mathbb {E}\Big [\dfrac{f_k(x_0) g_{k,j}(x_0) [t-s]}{[1\!+\!A(t,s)]^2} [W_j(t)-W_j(s)]\Big ] \;=\; 0\\&\mathbb {E}\Big [\Big <\dfrac{f(x_0)[t-s]}{1\!+\!A(t,s)}, \dfrac{g(x_0)}{1\!+\!A(t,s)} [W(t)-W(s)]\Big >_{\!\mathbb {R}^3}\Big ]\;=\; 0. \end{aligned}$$

### Proof

Consider

$$\begin{aligned}&\mathbb {E}\Big [\dfrac{f_k(x_0) g_{k,j}(x_0)}{[1\!+\!A(t,s)]^2} [W_j(t)-W_j(s)]\Big ]\\ =&\dfrac{1}{2}\left[ \mathbb {E}\Big [\dfrac{f_k(x_0) g_{k,j}(x_0)}{[1\!+\!A(t,s)]^2} [W_j(t)-W_j(s)]\Big ] + \mathbb {E}\Big [\dfrac{f_k(x_0) g_{k,j}(x_0)}{[1\!+\!A(t,s)]^2} [W_j(t)-W_j(s)]\Big ]\right] \\ =&\dfrac{1}{2}\left[ \mathbb {E}\Big [\dfrac{f_k(x_0) g_{k,j}(x_0)}{[1\!+\!A(t,s)]^2} [W_j(t)-W_j(s)]\Big ] + \mathbb {E}\Big [\dfrac{f_k(x_0) g_{k,j}(x_0)}{[1\!+\!A(t,s)]^2} [W_j(s)-W_j(t)]\Big ]\right] \\ =&\dfrac{1}{2}\left[ \mathbb {E}\Big [\dfrac{f_k(x_0) g_{k,j}(x_0)}{[1\!+\!A(t,s)]^2} [W_j(t)-W_j(s)]\Big ] - \mathbb {E}\Big [\dfrac{f_k(x_0) g_{k,j}(x_0)}{[1\!+\!A(t,s)]^2} [W_j(t)-W_j(s)]\Big ]\right] \\ &\;=\; 0 \end{aligned}$$

as the increments of $W_j$ are Gaussian distributed (symmetric), the increments $W_j(t)-W_j(s)$ are independent from $x_0=(s_0,i_0,r_0) \!\in \! \mathbb {D}$ appearing in *A*(*t*, *s*), the random variables *A*(*t*, *s*) do not change their sign and $f_k(x_0), g_{k,j}(x_0)$ are nonrandom. Therefore, we may compute

$$\begin{aligned} \mathbb {E}\Big [\Big <\dfrac{f(x_0)[t-s]}{1\!+\!A(t,s)},&\dfrac{g(x_0)}{1\!+\!A(t,s)} [W(t)-W(s)]\Big >_{\!\mathbb {R}^3}\Big ]\\ =&\sum _{k=1}^3 \sum _{j=1}^2 \underbrace{\mathbb {E}\Big [\dfrac{f_k(x_0) g_{k,j}(x_0)[t-s]}{[1\!+\!A(t,s)]^2} [W_j(t)-W_j(s)]\Big ]}_{\displaystyle = 0} \;=\; 0. \end{aligned}$$

$$\square $$

## Appendix D Comments on Standard SIR Models and Choices of Diffusion Contact Rates

So far, there is no agreement on the notion of "standard stochastic SIR model". However, most of the stochastic SIR models, which we have found in the literature, refer to the special case

$$ F_1(S,I,R) = \dfrac{K_1}{K_0+\delta _1 S^{\alpha _1} + \delta _2 I^{\alpha _2}} \quad \hbox {and} \quad F_2(S,I,R) = 0 $$

of diffusion contact rate functions $F_j$, where $K_j, \delta _i, \alpha _i \ge 0$ are non-negative real constants with $K_0>0$. For example, Guo et al. (2018) confine their model class to the choice $F_1(S,I,R)=\sigma / (1+\delta I^2)$ with positive constants $\delta \ge 0$. This choice is motivated by the classic choice of **Michaelis-Menton saturation law in population growth** in mathematical biology. Liu and Wang (2023) construct and verify a 1.5 order predictor-corrector numerical method for the SIS model confined to constant $F_1(S,I)=\sigma$ and $F_2(S,I)=0$, but staying on $\mathbb {D}=(0,N)^2$. They do not address what to do with more general functions $F_j$. Yang and Huang (2024) use a logarithmic transformation to obtain positivity preserving numerical methods for stochastic SIS model, restricted to a very specific case of $F_1(S,I)$ and $F_2(S,I)=0$. All those functions $F_j$ satisfy our key assumptions on the bounded domains $\mathbb {D}$ in our paper. Hence, they represent a special case of our models. But, we are also entitled to apply our results to their models. Moreover, our model class (1) with general diffusion rate functions $F_j$ is obviously much more general than the aforementioned models of other authors and it allows compartmental modeling "with true environmental variability" expressed by non-parametrically constrained functions $F_j$ on uniformly bounded domains $\mathbb {D}$.

Since $F_2$ enters in the dynamic equation of population *R* in our SIR model (1), more care is needed to guarantee that the random models exclusively stay on the uniformly bounded domain $\mathbb {D}$. The uniform boundedness of domains $\mathbb {D}$ in all variables is essential for adequate, biologically relevant models as all compartments are subject to limited, finite resources. The basic assumption for convergence proofs of our numerical methods has imposed the conditions that $\sup _{(S,I,R) \in \mathbb {D}} \frac{|F_2(S,I,R)|}{R} < +\infty$. Conditions like that are essential for guaranteeing that the solutions (*S*, *I*, *R*) stay forever in uniformly bounded domains $\mathbb {D}\subset \mathbb {R}^3_+$ (i.e., the invariance w.r.t. $\mathbb {D}$, especially the positivity of *R* at all times, despite random diffusion in its dynamic component). Most of the other publications relate to the case of unbounded domains such as $\mathbb {R}^3_+$. Thus they only refer to positivity, but allow unbounded random outcomes of at least one of the compartments *S*, *I*, *R*. That is in sharp contrast to the reality of finite resources for biological populations on earth.

For the most biologically meaningful choice of contact diffusion rates $F_2$ in the SIR model (1) we recommend to choose

$$ F_2(S,I,R) = \sigma _R \dfrac{R}{1+\delta _1 S^{\alpha _1} +\delta _2 I^{\alpha _2} +\delta _3 R^{\alpha _3}+\delta _4 [S+I +R]^{\alpha _4}} $$

with nonnegative constants $\sigma _R, \alpha _i, \delta _i \ge 0$. This form would model the intensity of a possibility of re-infection of a portion of the recovered or recovering population *R* by getting in contact with the infected population *I* again and getting back to the recovering compartment *R* of the total population $N=S+I+R$. So, this is seen as a substantial extension to apply it to the biologically relevant SIR models (1) (as the term $I F_2(S,I,R)$ enters in the diffusion components of *I* and *R* in (1)). Obviously, that form of $F_2$ satisfies our major convergence assumption

$$\sup _{(S,I,R) \in \mathbb {D}} \frac{|F_2(S,I,R)|}{R} \le |\sigma _R| < +\infty .$$

Hence, Theorem 10 on the global convergence of (12) to the solutions (*S*, *I*, *R*) of (1) can be applied to mathematically justify our BIMs (12) leaving the uniformly bounded domain $\mathbb {D}$ of the form (2) invariant for all paths; delivering stable, locally and globally consistent numerical outputs on $\mathbb {D}$, and being globally mean square convergent with guaranteed order $r_g=0.5$ on $\mathbb {D}$.

For the most biologically meaningful choice of contact diffusion rates $F_{\textrm{2 }}$in the SIR model (1) we recommend to choose

$$ F2(S,I,R) = \sigma R \frac{R}{1 + \delta _{\textrm{1}}S^{\alpha 1 }+ \delta _ 2 I^{\alpha 2} + \delta _3 R^{\alpha 3} + \delta _4[S + I + R]^{\alpha 4}} $$

with nonnegative constants $\sigma _{R},\alpha _{i},\delta _{i }\ge$0. This form would model the intensity of a possibility of re-infection of a portion of the recovered or recovering population *R*by getting in contact with the infected population *I*again and getting back to the recovering compartment *R*of the total population $N = S +$ $I + R$. So, this is seen as a substantial extension to apply it to the biologically relevant SIR models (1) (as the term $IF_{\textrm{2}}(S,I,R)$ enters in the diffusion components of *I*and *R*in (1)). Obviously, that form of $F_{\textrm{2 }}$satisfies our major convergence assumption

$$ \mathop {\text {sup}}\limits _{(S,I,R)\in \mathbb {D}} \frac{|F_{{2}}(S,I,R)|}{R} \le \sigma _{R}{<}+\infty . $$

Hence, Theorem 10 on the global convergence of (12) to the solutions (*S*, *I*, *R*) of (1) can be applied to mathematically justify our BIMs (12) leaving the uniformly bounded domain $\mathbb {D}$ of the form (2) invariant for all paths; delivering stable, locally and globally consistent numerical outputs on $\mathbb {D}$, and being globally mean square convergent with guaranteed order $r_{g }=$ 05 on $\mathbb {D}$.
